# Design, synthesis and antitumour activity evaluation of novel dolutegravir derivatives

**DOI:** 10.3389/fphar.2023.1238587

**Published:** 2023-08-07

**Authors:** Xi-Xi Hou, Long-Fei Mao, Yajie Guo, Chaoxuan Lou, Lan Wang, Rui-Fang Li, Huili Wang, San-Qiang Li, Jian-Xue Yang

**Affiliations:** ^1^ Department of Pharmacy, The First Affiliated Hospital, and College of Clinical Medicine of Henan University of Science and Technology, Luoyang, China; ^2^ College of Basic Medicine and Forensic Medicine, Henan University of Science and Technology, Luoyang, China; ^3^ Department of Emergency, The Eighth Affiliated Hospital, Sun Yat-Sen University, Shenzhen, China; ^4^ University of North Carolina Hospitals, Chapel Hill, NC, United States

**Keywords:** dolutegravir, 1,2,3-triazole, antitumor, autophagy, DNA damage

## Abstract

Based on the modification of the structure of dolutegravir, we introduced 1,2,3-triazole moieties with different substituted groups and obtained a lot of novel dolutegravir derivatives. The activity of A549 cells treated with the derivatives was examined, and most compounds showed good inhibitory effects. Among them, compounds **4b** and **4g** were the most effective, and inhibited the growth of A549 cells with IC_50_ values of 8.72 ± 0.11 μM and 12.97 ± 0.32 μM, respectively. In addition, compound **4g** induced apoptosis and clonal suppression in A549 tumor cells. Compound **4g** also activated the LC3 signaling pathway to induce autophagy in tumor cells, and activated the γ-H2AX signaling pathway to induce DNA damage in tumor cells.

## 1 Introduction

**FIGURE 1:**
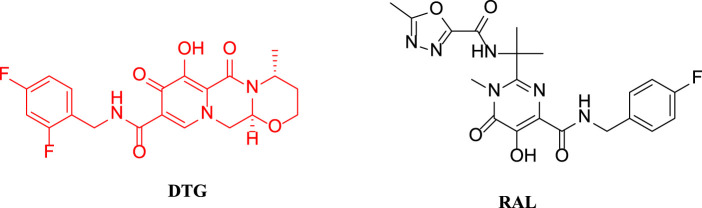
The structures of DTG and RAL.

Dolutegravir (DTG, Figure 1) is an HIV integrase inhibitor that blocks the strand transfer step of retroviral DNA integration by binding to the active site of the integrase. An *in vitro* experiment found that dolutegravir inhibited the strand transfer catalyzed by recombinant HIV-1 integrase with a half maximal inhibitory concentration (IC_50_) of 2.7 nM(Johns et al., 2013; Jay et al., 2020; Manoj et al., 2020). Patients only take this drug once daily; among patients infected with HIV-1 for the firsttime, the therapeutic effect of dolutegravir is equivalent to that of raltegravir (RAL, Figure 1), which is administered twice per day (Eron et al., 2013). Furthermore, dolutegravir possesses potent anti-resistance properties. Preclinical study results showed that dolutegravir had low toxicity and no genotoxicity or carcinogenic toxicity, and there was no apparent teratogenicity or reproductive toxicity when the dose was 27-fold greater than the clinical dose (Chen et al., 2018). Dolutegravir has been combined with other antiretroviral drugs, such as lamivudine or abacavir, to form highly active antiretroviral therapy (ART), which has been recommended by the WHO as the first-line treatment drug for all populations, including pregnant women and women of childbearing potential (Collins et al., 2021).

**FIGURE 2:**
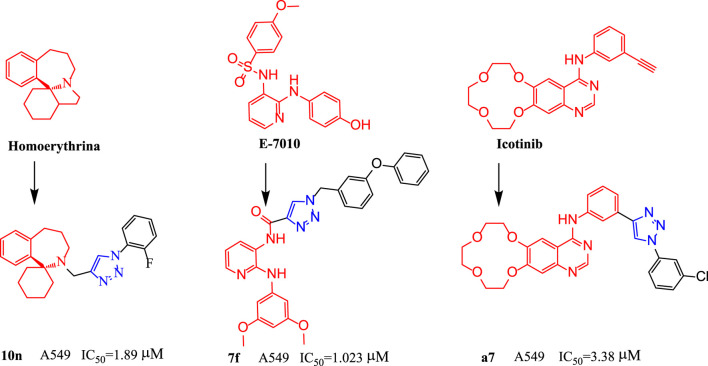
The structures of compounds **10n**, **7f** and **a7**.

With the high therapeutic effect of ART, HIV-related opportunistic infections have been effectively controlled. However, with a prolonged disease course, patients are in a long-term immunosuppressive state, and malignant tumors have increasingly become the main cause of death in these patients. Some studies found that among young HIV-infected patients, the primary lung cancer incidence was more than six-fold that of the general population (Biggar Robert et al., 2007; Piketty et al., 2012). As a result, we attempted to modify the molecular structure of the HIV integrase inhibitors for anti-tumor activity. Due to its relatively strong anti-HIV activity and good safety and tolerability, dolutegravir was investigated in this study. In our study, we introduced 1,2,3-triazole groups into the structure of dolutegravir by click reaction. 1,2,3-Triazole is an important nitrogen-containing heterocyclic compound. Because1,2,3-triazole has amide isosteres and a stable rigid plane and can be prepared efficiently using a click reaction, it has been widely used for the modification of drug molecules, especially for the development of novel anti-tumor drugs (Wu et al., 2017; Sainas et al., 2019; Madasu et al., 2020; Pan et al., 2020; Tangadanchu et al., 2020). For example, as show in Figure 2, one research group synthesized a series of homoerythrina alkaloid derivatives containing 1,2,3-triazole. Among them, compound **10n** showed a relatively strong inhibitory effect on A549 cells (IC_50_ = 1.89 μM), which was stronger than harringtonine (IC_50_ = 10.55 μM), pemetrexed (IC_50_ = 3.39 μM), and rucaparib (IC_50_ = 4.91 μM). Compound **10n** effectively arrested the cell cycle at the S phase, thus inducing apoptosis (apoptosis rate: 46%) and effectively inhibiting cell proliferation (Li et al., 2020).The research group led by Kamal modified the pyridine-sulfonamide derivative E7010 by replacing the benzenesulfonic acid structure with a 1,2,3-triazole moiety. Among the products, compound **7f** showed a strong inhibitory effect on A549 cells (IC_50_ = 1.023 μM), and in addition, it arrested the cell cycle at the G2/M phase, thus inducing apoptosis of A549 cells. Detection of the mitochondrial membrane potential further confirmed the induction of apoptosis by compound **7f**. Molecular docking studies indicated that compound **7f** targeted the colchicine site of *β*-tubulin, and the mode of action was similar to that of E7010. Furthermore, the inhibitory effect of **7f** on *β*-tubulin (IC_50_: 2.04 µM) was equivalent to that of E7010 (IC_50_ = 2.15 µM) (Prasad et al., 2019). Because the epidermal growth factor receptor (EGFR) inhibitor icotinib has a structural feature of a terminal alkyne, it reacted with 3-chlorophenyl azide to produce compound **a7**, which showed excellent inhibitory effects on mutant lung cancer cells (PC-9) and wild-type lung cancer cells (A549), and had a stronger effect than icotinib (Mao et al., 2020). Compound **a7** downregulated the expression of caspase-3, causing fragmentation, pyknosis, and dense hyperchromasia of nuclei in A549 cells, inducing apoptosis and arresting A549 cells at the G2/M phase.

Therefore, a series of 1,2,3-triazoles derivatives were designed and synthesized by click reaction using dolutegravir as the parent nucleus according to the principle of bioactive substructure splicing. We used CCK-8 method to evaluate the anti-proliferative activity of the target compounds on lung carcinoma cell line A549.

## 2 Chemistry

**FIGURE 3:**
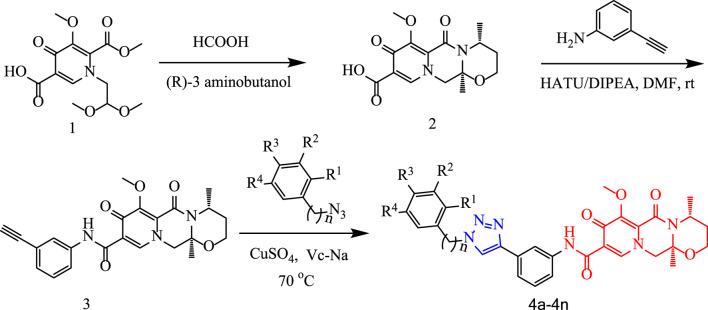
The reaction routes to compounds **4a**-**4n**.

In this route, 1-(2,2-dimethoxyethyl)-5-methoxy-6-(methoxycarbonyl)-4-oxo-1,4-dihydropyridine-3-carboxylic acid (**1**) was used as raw material and it was hydrolyzed under formic acid. The *(R)*-3 aminobutanol was added directly to the vacuum concentration and the mixture was refluxed in acetonitrile to give compound **2**. Compound **2** was condensed with 3-amine phenylacetylene to obtain terminal alkyne compound **3**. Compound **3** was reacted with azide compounds of different substituents to obtain 14 novel structure target compounds **4a-4n** as shown in Figure 3 and Table 1. The structures of the target compound were confirmed through ^1^H and ^13^C nuclear magnetic resonance spectroscopy.

**TABLE 1:** R-group of compounds 4a-4n.

Compd no.	n	R_1_	R_2_	R_3_	R_4_	Compd no.	n	R_1_	R_2_	R_3_	R_4_
4a	0	F	H	H	H	4h	0	CF_3_	H	H	CF_3_
4b	0	CH_3_	NO_2_	H	H	4i	0	OCH_3_	H	H	H
4c	0	H	CH_3_	H	H	4j	0	Br	H	H	H
4d	0	H	H	F	H	4k	0	Cl	H	H	H
4e	0	CH_2_CH_3_	H	H	H	4L	0	I	H	H	H
4f	0	H	OCH_3_	H	H	4m	1	Br	H	H	H
4g	0	H	CF_3_	H	H	4n	1	H	Br	H	H

## 3 Results and discussion

### 3.1 Compounds 4a-4n suppressed cancer cells viability

In order to investigate the anti-proliferative activity of dolutegravir-1,2,3-triazole derivatives to lung carcinoma cell, we performed CCK8 assay to detect the effects of all the compounds on the cell viability of A549 cell line. We measured and calculated the half-maximal inhibitory concentration (IC_50_) of all the compounds. As showed in Table 2, **4b** and **4g** was suggested as the most highly active compound against A549 cell with IC_50_ values at 8.72 ± 0.11 and 12.97 ± 0.32 μM, respectively. LO2 and BESA-2b cells were a kind of normal cell lines which was used as a control to make a comparison with cancer cell lines and were treated with compounds **4b** and **4g** at the concentration of 20 μM for 48 h. The cell viabilities of LO2 cell for compounds **4b** and **4g** were 55.37% and 79.49%, and the cell viabilities of BESA-2b cell for compounds **4b** and **4g** were 61.57% and 76.42%.

**TABLE 2:** The half-maximal inhibitory concentration (IC_50_) of all the compounds.

Compd no.	IC_50_, µM, 48h	Compd no.	IC_50_, µM, 48h
	A549		A549
4a	>50	4h	13.63 ± 1.79
4b	8.72 ± 0.11	4i	17.34 ± 0.73
4c	27.75 ± 0.56	4j	>50
4d	>50	4k	>50
4e	20.67 ± 0.23	4L	33.66 ± 0.68
4f	44.34 ± 1.21	4m	>50
4g	12.97 ± 0.32	4n	>50
		DTG	>50

### 3.2 Compounds 4b and 4g inhibited proliferation of cancer cells

**FIGURE 4:**
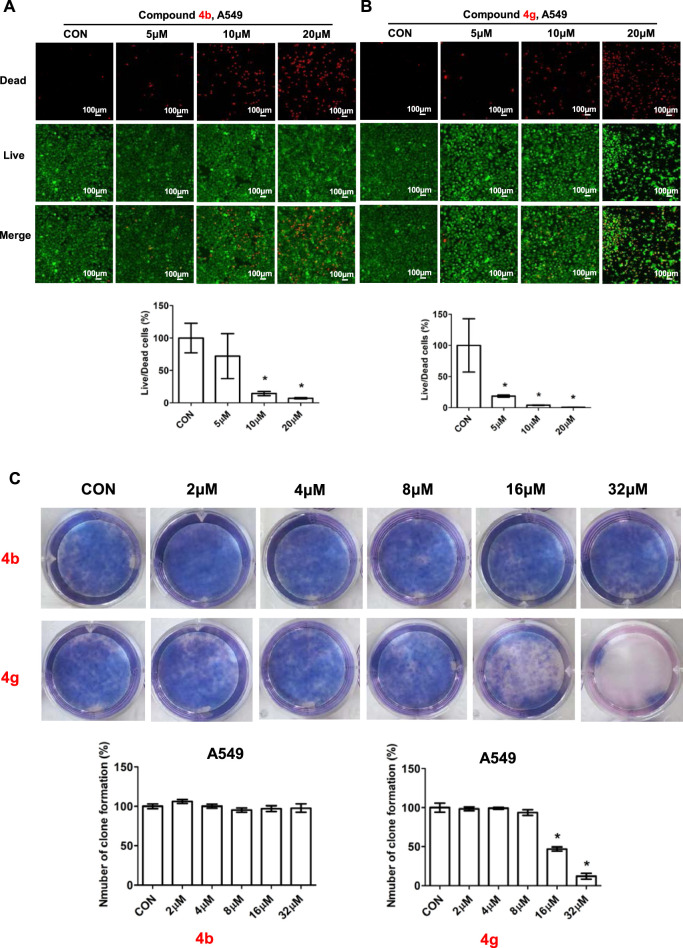
Compounds 4b and 4g inhibited proliferation of cancer cells. **(A)** Fluorescence images stained with the LIVE/DEAD kit of A549 cells treated with 5 μM, 10 μM, 20 μM of **4b**. **(B)** Fluorescence images stained with the LIVE/DEAD kit of A549 cells treated with 5 μM, 10 μM, 20 μM of **4g**. **(C)** Plate clone staining of A549 cells treated with different concentrations of **4b** and **4g**. Data are presented as mean ± SE. *p < 0.05.

To further assess the anti-proliferative activity of dolutegravir derivatives, we utilized LIVE/DEAD staining. Specifically, A549 cells were treated with 5 μM, 10 μM, or 20 μM concentrations of **4b** or **4g** for 24 h, and subsequently imaged and counted to distinguish between live and dead cells. As demonstrated in Figure 4, live A549 cells were significantly reduced in a dose-dependent manner following treatment with either **4b** or **4g**. Moreover, the ratio of dead/live cells was also found to increase substantially with increasing concentration.

To validate the impact of **4b** and 4g on cell proliferation, we employed a plate clone formation assay. In this assay, A549 cells were treated with varying concentrations (0, 2, 4, 8, 16, and 32 μM) of either **4b** or **4g**. Our results indicated that compound **4b** had no discernible effect on the A549 cell colony formation experiment on the plate. In contrast, compound **4g** exhibited anti-proliferative activity in a dose-dependent manner across all cell lines tested, including A549.

### 3.3 Compounds 4b and 4g induced apoptosis of cancer cells

**FIGURE 5:**
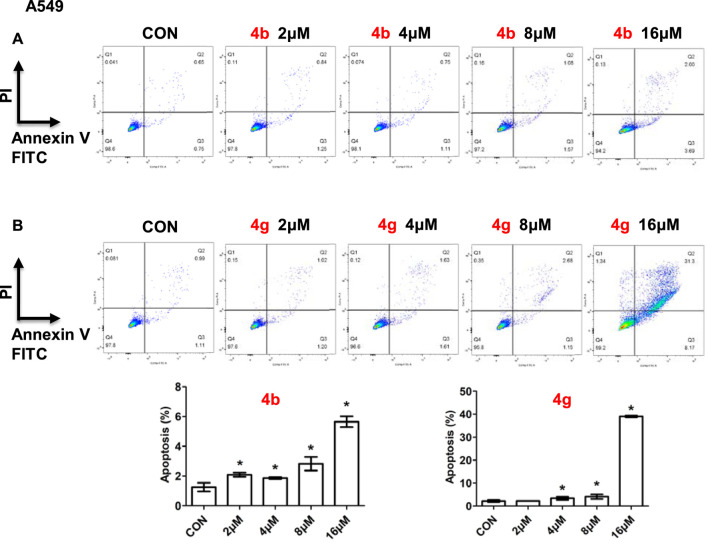
Compounds 4b and 4g induced apoptosis of cancer cells. Apoptotic cells of A549 cells treated with 4b **(A)** and 4g **(B)** determined by flow cytometry. Data are presented as mean ± SE. *p < 0.05.

Since dolutegravir derivatives could repress cancer cells proliferation, to explore whether they had effects on cell apoptosis, the apoptosis analysis was performed. A549 cells treated with different concentrations of **4b** or **4g** were stained with Annexin V-FITC and PI, and the numbers of apoptosis cells were analyzed by the flow cytometry. As illustrated, for A549 cells, compound **4b** showed little influence on cell apoptosis (Figure 5A). In addition, A549 cells treated with 16 μM of **4g** for 48h displayed a significant increase in the percentage of apoptosis but had no changes when treated with 2 μM, 4 μM or 8 μM of **4g** (Figure 5B).

### 3.4 Compounds 4b and 4g affected protein expressions of key signaling pathways

**FIGURE 6:**
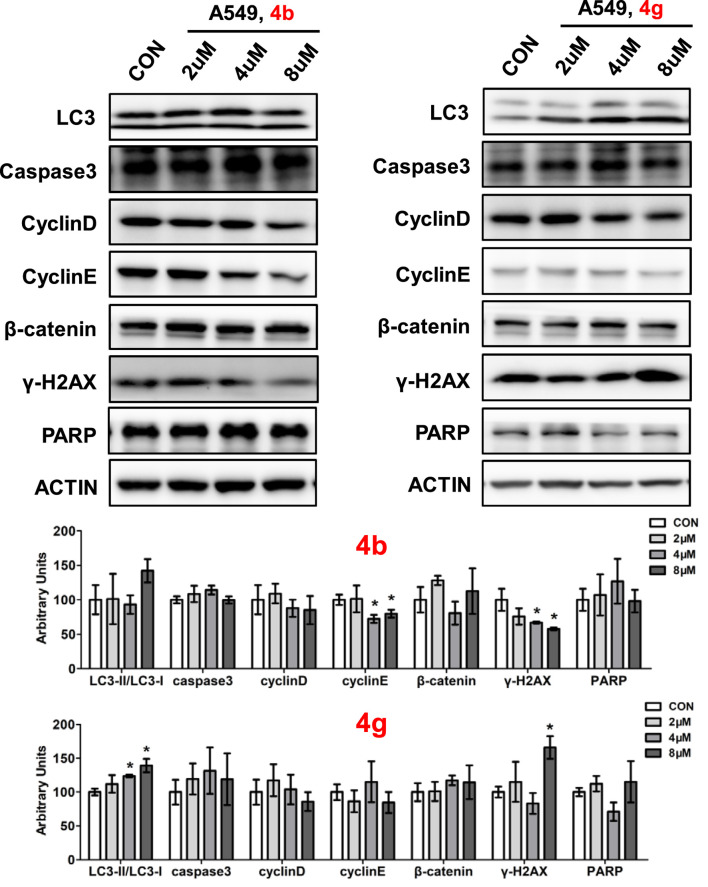
Compounds 4b and 4g affected protein expressions of key signaling pathways. Western blotting of LC3, caspase3, Cyclin D, Cyclin E, β-catenin, γ-H2AX, PARP in A549 cells treated with **4b** and **4g**. Top, Western blot; bottom, quantitative measurements relative to ACTIN. Data are presented as mean ± SE. *p < 0.05.

To determine the role of dolutegravir derivatives in regulating the development of cell proliferation, key proteins expressions which were involved in cell growth progresses were examined, including autophagy, apoptosis, cell cycle and DNA damage (Figure 6). Ubiquitin like molecule light chain 3 (LC3) is a key marker of autophagy, our results showed that the expression of LC3 was significantly increased after compound 4g treated while had no difference after compound 4b treated in A549 cells. Caspase3, one of the key proteins in regulating apoptosis, however, was not changed in cancer cells when added **4b** or **4g** (Figure 6). Cell cycle related genes including CyclinD, CyclinE or β-catenin also showed no differences with **4b** or **4g** treatment in A549 cells. In A549 cells, γ-H2AX was induced when treating with **4g** and PARP showed no differences with **4b** or **4g** treatment.

## 4 Conclusion

In this study, based on the modification of the structure of dolutegravir, we introduced 1,2,3-triazole moieties with different substituted groups and obtained 14 dolutegravir-1,2,3-triazole derivatives. The activity of A549 cells treated with the derivatives was examined, and most compounds showed strong inhibitory effects. Among them, compounds **4b** and **4g** were the most effective, and inhibited the growth of A549 cells with IC_50_ values of 8.72 ± 0.11 μM and 12.97 ± 0.32 μM, respectively. In addition, compound 4g induced apoptosis and clonal suppression in A549 tumor cells. Compound **4g** also activated the LC3 signaling pathway to induce autophagy in tumor cells, and activated the γ-H2AX signaling pathway to induce DNA damage in tumor cells. This research has guiding significance for the conversion of non-anti-tumor clinical drugs into lead compounds with anti-tumor activity.

## 5 Experimental

### 5.1 Materials and chemistry

The dolutegravir-1,2,3-triazole derivative was synthesised in-house. All the reagents and solvents used were obtained from a commercially available source. The ^1^H and ^13^C NMR spectra were acquired in a DMSO-*d*_*6*_ solution using a Bruker 400 MHz or 600 MHz NMR spectrometer. LC-MS instrument was carried out using a Waters ZQ 2000. Dulbecco’s modified Eagle medium (DMEM), RPMI 1640 Medium, Fetal bovine serum (FBS) and penicillin/streptomycin were purchased from Gibco (Grand Island, NY, United States).Enhanced Cell Counting Kit-8, Calcein/PI Live/Dead Viability Assay Kit and Giemsa dye were obtained from Beyotime Biotechnology (Shanghai, China). Annexin V-FITC/Propidium iodide (PI) staining kit and Matrigel Matrix were provided by BD Biosciences (Franklin Lake, New Jersey, United States).

#### 5.1.1 Synthesis of (4R,12aS)-3,4,6,8,12,12a-hexahydro-7-methoxy-4-methyl-6,8-dioxo-2H-pyridine [1′,2′: 4,5]pyrazino[2,1-b][1,3]oxazine-9-carboxylic acid (Compound 2)


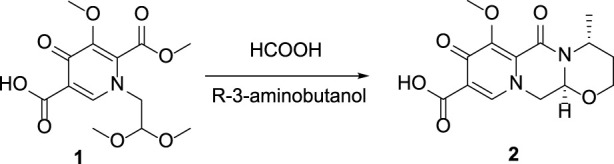


In a reaction flask, 1-(2,2-dimethoxyethyl)-1,4-dihydro-3-methoxy-4-oxo-2,5-pyridinedicarboxylic acid-2-methyl ester (compound **1**, 30 g, 0.1 mol) was added to 150 mL of anhydrous formic acid. The reaction was carried out at 65°C with stirring and under argon atmosphere. The reaction was completed at about 3 h when the starting material was used up as monitored with TLC. Under the vacuum, concentrated and evaporate formic acid at 45°C to give a crude oil. Add 150 mL of acetonitrile to the crude oil to dissolve with stirring. Added R-3-aminobutanol (12.5 g, 0.14 mol) and stirred for 10 min. Then the temperature was raised to an internal temperature of 82°C and continue stir for 2 h. The reaction completed at this time as monitored with TLC. Concentrate to remove most of the solvent at 45°C. Added 200 mL of dichloromethane, and then 100 mL water while stirring. Used 2N HCl to adjust the pH to 1-2, stir for 10 min, then separated the lower organic phase. The upper aqueous phase was extracted three times with 50 mL of dichloromethane. Combined all organic phases and washed three times with 50 mL of saturated NaCl solution. Concentrated the mixture under vacuum to give a crude product. It was then purified by recrystallization using methanol to gave 21 g of pure product (Compound **2**), yield 68.7%; ^1^H NMR (400 MHz, CDCl_3_): δ 8.43 (s, 1H), 5.30 (t, J_1_ = 4.0 Hz, J_2_ = 4.0 Hz, 1H), 5.02 (t, J_1_ = 4.0Hz, J_2_ = 8.0Hz, 1H), 4.41 (dd, J_1_ = 4.0Hz, J_2_ = 4.0Hz, 1H), 4.27 (dd, J_1_ = 8.0Hz, J_2_ = 4.0Hz, 1H), 4.08 (s, 3H), 4.03–3.99 (m, 2H), 2.25–2.16 (m, 1H), 1.56 (d, J = 12.0Hz, 1H), 1.39 (d, J = 8.0Hz, 3H). ^13^C NMR (100 MHz, CDCl_3_): δ 176.39, 165.85, 155.00, 153.90, 142.78, 130.66, 116.08, 75.97, 62.65, 61.48, 53.89, 44.93, 29.37, 16.06.

#### 5.1.2 General synthetic procedure for compound 3


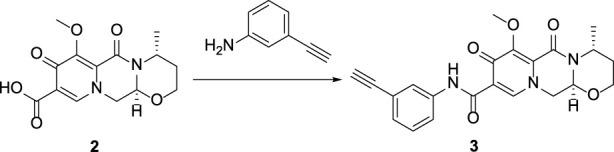


Compound **2** (5 g), 3-aminophenylacetylene (3.69g), HATU (13 g), DIPEA (8.2 g) and solvent DMF 250 mL were added to a 500 mL reaction flask at room temperature and stirred under nitrogen protection for 24 h. Thin layer chromatography (TLC) was used for monitoring. After 24 h, the reaction completed and the reaction solution was light brown. DMF was removed by vacuum concentration, dichloromethane was added to extract the reaction solution (150 mL × 3).Combine all organic solutions, wash them with saturated sodium chloride (150 mL × 2) to pH = 7, and the viscous brownish yellow liquid was obtained by vacuum distillation. Under ultrasonic vibration, methanol was slowly added drop by drop, and solid precipitated. After that, it was left to stand, filtered and dried to obtain the compound **3**, 4.7 g, yield 71.2%.

#### 5.1.3 General synthetic procedure for compounds 4a-4n


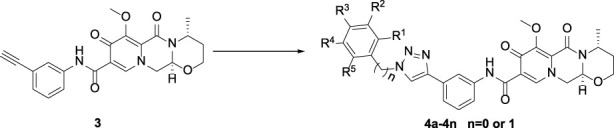


In the reaction flask, compound **3** (3 mmol), substituted azide (3.6 mmol), TERT butanol 70 mL, water 70 mL, tetrahydrofuran 70 mL, anhydrous copper sulfate (1.2 g, 6 mmol) and sodium ascorbate (0.36 g, 1 mmol) were successively added, and stirred and refluxed at 70°C for 6 h. After the reaction was completed (monitored by TLC), use dichloromethane (100 mL × 3) to extract, combine the organic solution and wash with saturated sodium chloride aqueous solution (100 mL × 2). The combined organic layer was washed with brine (100 mL × 2), dried over sodium sulfate, and concentrated *in vacuo* to give the crude product. Recrystallization in ethyl acetate produced the desired compound which was pure enough for further characterization and anti-tumor study.

The spectroscopic characterization of compounds **4a**-**4n** is provided as Supporting Material Data.

### 5.2 Biological study

#### 5.2.1 Cell culture

Human lung cancer cell lines A549 was obtained from ATCC. Cells were cultured in DMEM or RPMI 1640 medium containing 10% FBS and 1% penicillin/streptomycin at 37 °C with a 5% CO_2_-humidified atmosphere.

#### 5.3.2 Cell viability assay

CCK8 assay was used to measure cell viability. Cells with a density of 1 × 10^4^ cells/well were seeded on the 96-well plates. After adhesion, cells were treated with different diluted compounds or vehicle control DMSO and continue cultured for 48 h respectively. Then, CCK8 reagent was added for 1 hour incubation at 37°C with 5% CO_2_. Absorbance was measured using a Microplate spectrophotometer (Thermo) at 450 nm. The ratio of cell viability of control was taken as 100%. For IC_50_, cells were treated with different concentrations of compounds (0, 0.5, 2, 8, 16, 32 μM) for 48 h and cell viability was determined to calculate the inhibition percentage. The CCK-8 assay was conducted three times and the repetitions in each time were at least three. Then IC_50_ of compounds were investigated using the prism statistical software.

#### 5.3.3 Live and dead cells measurement

A549 cells with a density of 5 × 10^3^ cells/well were seeded on the 96-well plates. Then different concentrations (0, 5, 10, 20 μM) of **4b** or **4g** were treated for 24 h. Cells were then stained with the LIVE/DEAD Assay Kit, observed and photographed using the fluorescent microscope.

#### 5.3.4 Plate clone formation assay

A549 cells were seeded into 6-well plates at a density of 200–500 cells/well. After 10 days culture, cells were added with **4b** or **4g** at different concentrations (0, 2, 4, 8, 16, 32 μM) for 48 h. Then cells were fixed by 4% paraformaldehyde and stained by Giemsa dye. An optical microscope was used to photographed cells and counted the clone numbers.

#### 5.3.5 Apoptosis assay

A549 cells were cultured in 6-well plates with a density of 3 × 10^5^ cells/well. Different concentrations of 4b or 4g were added to cells for 48h respectively. The concentrations were 0, 2, 4, 8, and 16 μM for A549 cells. After treatment, Annexin V-FITC Apoptosis Detection Kit was used to determine the apoptotic ratio and FlowJo software v10 was used to analyze.

#### 5.3.6 Western blot

Protein expression levels were measured by Western blot. A549 cells were cultured in 12-well plates and different concentrations (0, 2, 4, 8 μM) of **4b** and **4g** were added for 48h. Proteins were extracted from whole cells using radioimmunoprecipitation assay (RIPA) buffer containing protease/phosphatase inhibitor cocktail (CST). 10%–15% sodium dodecyl sulfate polyacrylamide gel electrophoresis and nitrocellulose membranes (Millipore) were used to separated and collected proteins. Antibodies used include, LC3 (3868s, CST), Caspase3 (9662, CST), cyclin D (2922s, CST), cyclin E (20808s, CST), γH2AX (9718s, CST), β-Catenin (9562s, CST), PARP (46D11, CST), and β-actin (4967s, CST).

#### 5.3.7 Statistical analyses

Data were conducted using Graph Prim 7.0.A two-tailed Student’s t-test or one-way analysis of variance followed by a Student-Newman-Keuls (SNK) test were used to assess significant differences. Values of *p* < 0.05 were considered statistically significant.

## Data Availability

The original contributions presented in the study are included in the article/Supplementary Materials, further inquiries can be directed to the corresponding author/s.
