# Supplementary material for: Design, synthesis and antitumour activity evaluation of novel dolutegravir derivatives
Source: Front Pharmacol. 2023 Aug 7;14:1238587. doi: 10.3389/fphar.2023.1238587 (PMC10440426; doi:10.3389/fphar.2023.1238587)

**Design, Synthesis and antitumour activity evaluation of novel dolutegravir derivatives**

Xi-xi Hou^a^, Long-fei Mao^a,b^, Yajie Guo^c^, Chaoxuan Lou^a^, Lan Wang ^b^, Rui-fang Li ^b^, Huili Wang^d*^, San-qiang Li ^b *^, Jian-xue Yang^a*^

^a^The First Affiliated Hospital, and College of Clinical Medicine of Henan University of Science and Technology, Luoyang, 471003, China

^b^College of Basic Medicine and Forensic Medicine, Henan University of Science and Technology, 263 Kaiyuan Road, Luoyang 471003, China

^c^Department of Emergency, The Eighth Affiliated Hospital, Sun Yat-Sen University, Shenzhen, 518033, China

^d^University of North Carolina Hospitals, 101 Manning Dr, Chapel Hill, Orange County, NC 27599, USA

†These authors have contributed equally to this work as first co-authors

*Corresponding authors:

**Huili Wang**, UNC Hospital, 101 Manning Dr. Chapel Hill, NC 27599, USA. E-mail: huili.be@gmail.com

**San-qiang Li,** College of Basic Medicine and Forensic Medicine, Henan University of Science and Technology, 263 Kaiyuan Road, Luoyang 471003, China. E-mail: sanqiangli2001@163.com

**Jian-xue Yang**, The First Affiliated Hospital, and College of Clinical Medicine of Henan University of Science and Technology, Luoyang, 471003, China. E-mail: Docyix1969@126.com

**Compound** 4a：Pure 98.9%. [white solid](javascript:;), HR-MS(ESI): Calcd. C28H25FN6O5 [M+H]^+^ *m/z*: 545.1949, found: 545.1988. m.p. 192-195 ^o^C. ^1^H NMR (400MHz, DMSO-d_6_): 12.62 (s, 1H), 9.17 (s, 1H), 8.73 (s, 1H), 8.24 (s, 1H), 7.90 (d, J=24.0Hz, 2H), 7.68 (d, J=16.0Hz, 3H), 7.50 (s, 2H), 5.40 (s, 1H), 4.80 (s, 1H), 4.65 (s, 1H), 4.43 (s, 1H), 3.98 (s, 1H), 3.89-3.86 (m, 4H), 1.98 (s, 1H), 1.54 (s, 1H), 1.30 (d, J=8.0Hz, 3H). ^13^C NMR (100MHz, DMSO-d_6_): 174.27, 162.21, 155.65, 153.49, 153.25, 147.12, 143.60, 139.50, 131.32, 130.86, 130.29, 126.47, 126.09, 123.59, 121.41, 120.03, 118.13, 117.75, 117.62, 116.95, 76.31, 62.23, 60.71, 55.38, 52.86, 44.83, 29.67, 16.18.


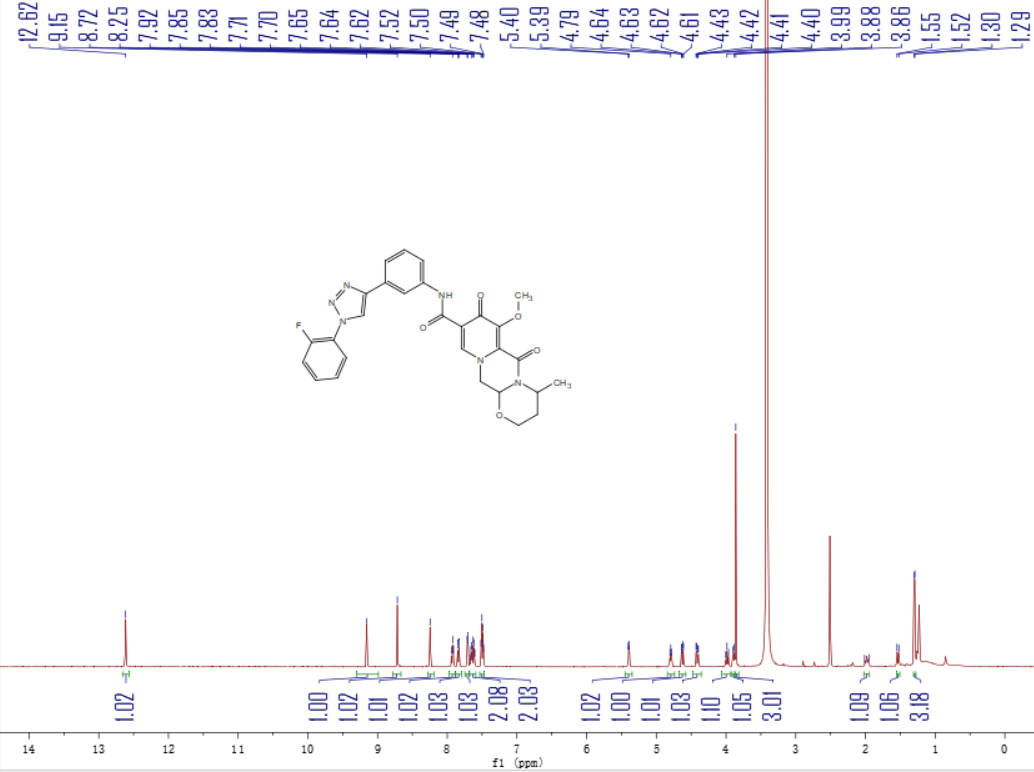


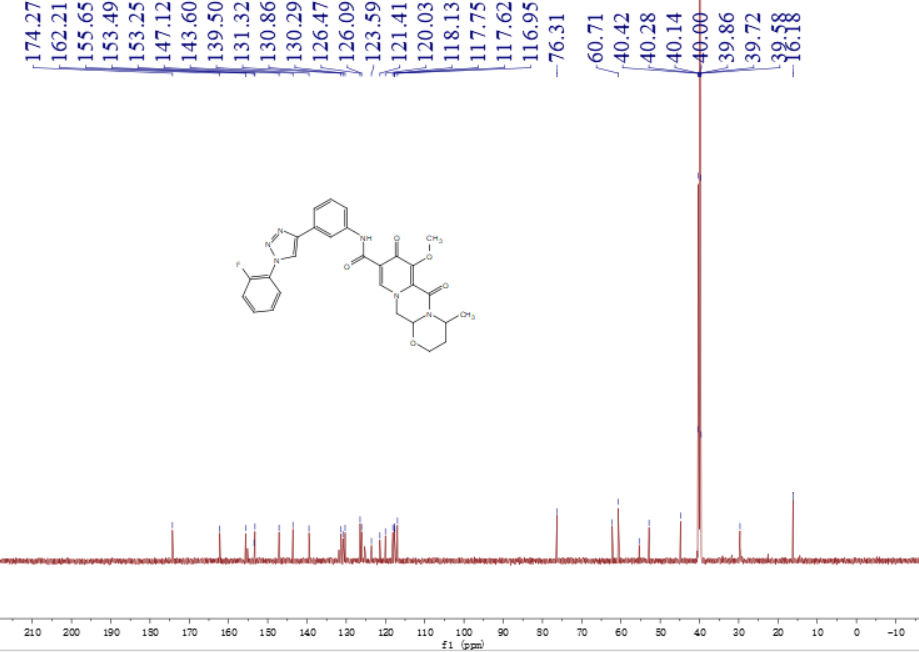


**Compound** 4b：Pure 98.2%. [yellow solid](javascript:;), HR-MS(ESI): Calcd. C29H27N7O7 [M+H]^+^ *m/z*: 586.2050, found: 586.2063. m.p. 178-181 ^o^C. ^1^H NMR (400MHz, DMSO-d_6_): 12.63 (s, 1H), 9.12 (s, 1H), 8.71 (s, 1H), 8.20 (t, *J_1_*=8.0Hz, *J_2_*=8.0Hz, 2H), 7.95 (d, *J*=8.0Hz, 1H), 7.84 (d, *J*=8.0Hz, 1H), 7.73 (dd, *J_1_*=8.0Hz, *J_2_*=4.0Hz, 2H), 7.50 (t, *J_1_*=8.0Hz, *J_2_*=8.0Hz, 1H), 5.39 (s, 1H), 4.79 (s, 1H), 4.62 (d, *J*=12.0Hz, 1H), 4.41 (d, *J*=8.0Hz, 1H), 3.98 (t, *J_1_*=12.0Hz, *J_2_*=12.0Hz, 1H), 3.89 (s, 1H), 3.85 (s, 3H), 2.26 (s, 3H), 1.97 (s, 1H), 1.53 (d, *J*=12.0Hz, 1H), 1.29 (d, *J*=8.0Hz, 3H)；^13^C NMR (100MHz, DMSO-d_6_): 173.77, 161.70, 155.15, 152.74, 150.78, 146.48, 143.08, 139.00, 137.63, 130.95, 130.86, 130.36, 129.82, 128.19, 128.00, 125.60, 123.99, 120.88, 119.54, 117.62, 116.43, 75.80, 61.72, 60.21, 52.36, 44.35, 29.17, 15.68, 13.96.


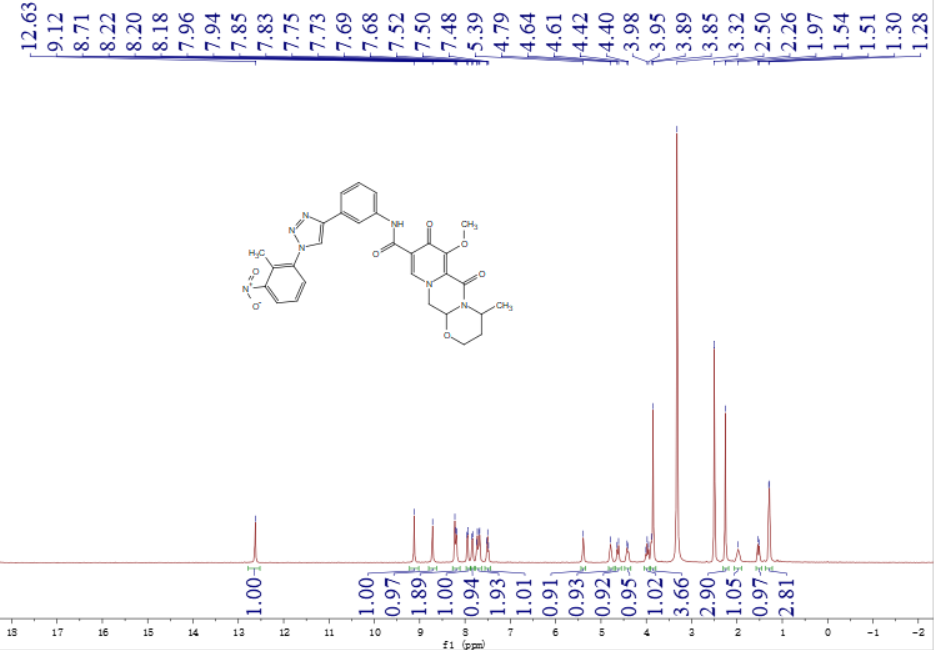


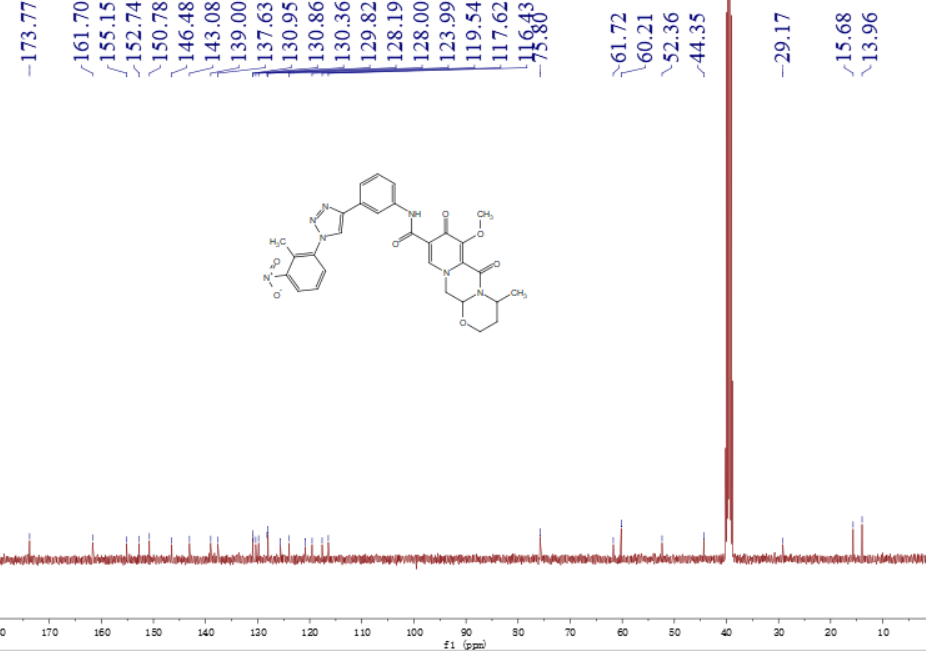


**Compound** 4c：Pure 98.4%. [white solid](javascript:;), HR-MS(ESI): Calcd. C29H28N6O5 [M+Na]^+^ *m/z*: 563.2019, found: 563.2067. m.p. 148-151 ^o^C. ^1^H NMR (400MHz, DMSO-d_6_): 12.63 (s, 1H), 9.36 (s, 1H), 8.72 (s, 1H), 8.18 (s, 1H), 7.88 (d, *J*=8.0Hz, 1H), 7.84 (s, 1H), 7.78 (d, *J*=8.0Hz, 1H), 7.71 (d, *J*=8.0Hz, 1H), 7.50 (dd, *J_1_*=8.0Hz, *J_2_*=8.0Hz, 2H), 7.34 (d, *J*=4.0Hz, 1H), 5.41-5.39 (m, 1H), 4.83-4.77 (m, 1H), 4.66-4.62 (m, 1H), 4.45-4.40 (m, 1H), 3.99 (t, *J_1_*=12.0Hz, *J_2_*=12.0Hz, 1H), 3.90 (s, 1H), 3.87 (s, 3H), 2.46 (s, 3H), 2.05-1.94 (m, 1H), 1.53 (d, *J*=12.0Hz, 1H), 1.30 (d, *J*=8.0Hz, 3H). ^13^C NMR (100MHz, DMSO-d_6_): 174.30, 162.20, 155.67, 153.27, 147.43, 143.59, 140.17, 139.50, 137.09, 131.58, 130.87, 130.29, 130.21, 129.78, 121.29, 120.86, 120.28, 119.96, 118.15, 117.53, 116.86, 76.31, 62.25, 60.73, 52.89, 44.86, 29.69, 21.43, 16.20.


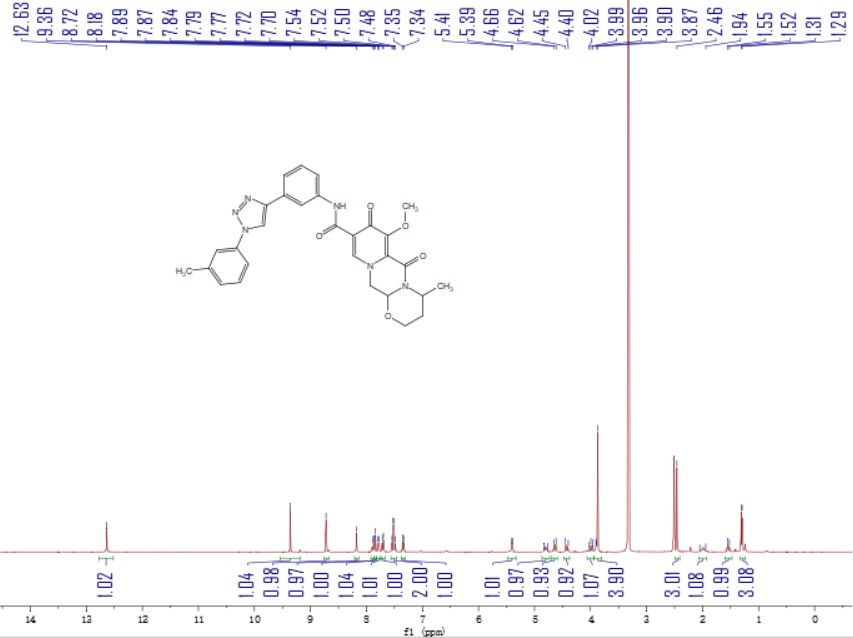


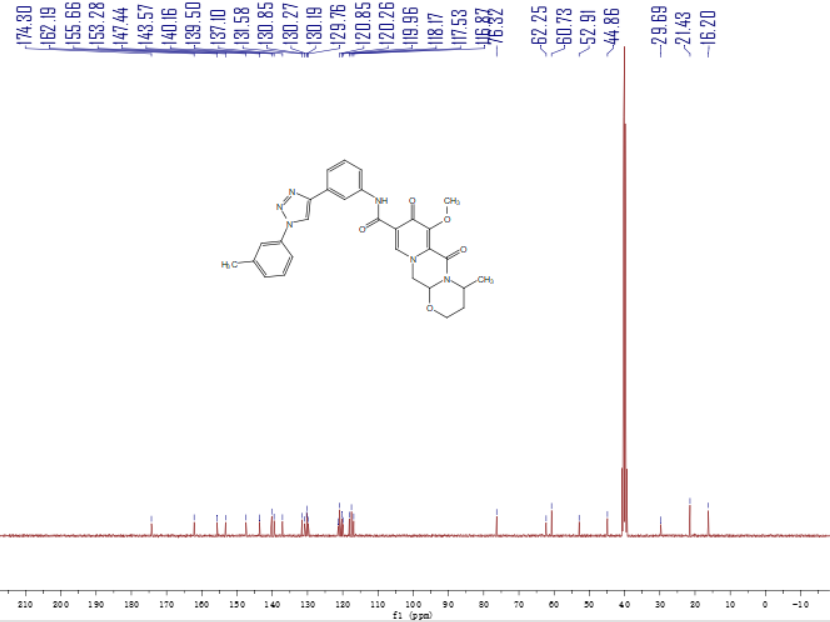


**Compound** 4d：Pure 97.2 %. [white solid](javascript:;), HR-MS(ESI): Calcd. C28H25FN6O5 [M+H]^+^ *m/z*: 545.1949, found: 545.1997. m.p. 201-204 ^o^C. ^1^H NMR (400MHz, DMSO-d_6_): 12.63 (s, 1H), 9.36 (s, 1H), 8.71 (s, 1H), 8.17 (s, 1H), 8.03 (dd, *J_1_*=4.0Hz, *J_2_*=4.0Hz, 2H), 7.87 (d, *J*=8.0Hz, 1H), 7.69 (d, *J*=8.0Hz, 1H), 7.50 (t, *J_1_*=8.0Hz, *J_2_*=8.0Hz, 3H), 5.39 (s, 1H), 4.79 (t, *J_1_*=4.0Hz, *J_2_*=8.0Hz, 1H), 4.62 (d, *J*=12.0Hz, 1H), 4.42 (dd, *J_1_*=8.0Hz, *J_2_*=8.0Hz, 1H), 3.98 (t, *J_1_*=12.0Hz, *J_2_*=12.0Hz, 1H), 3.89 (s, 1H), 3.86 (s, 3H), 2.01-1.94 (m, 1H), 1.52 (d, *J*=12.0Hz, 1H), 1.29 (d, *J*=8.0Hz, 1H). ^13^C NMR (100MHz, DMSO-d_6_): 174.31, 162.20, 155.65, 153.28, 147.54, 143.58, 139.50, 131.50, 130.85, 130.30, 122.86, 122.78, 121.30, 120.61, 120.01, 118.16, 117.37, 117.14, 116.88, 76.32, 62.25, 60.73, 52.91, 44.87, 29.69, 16.20


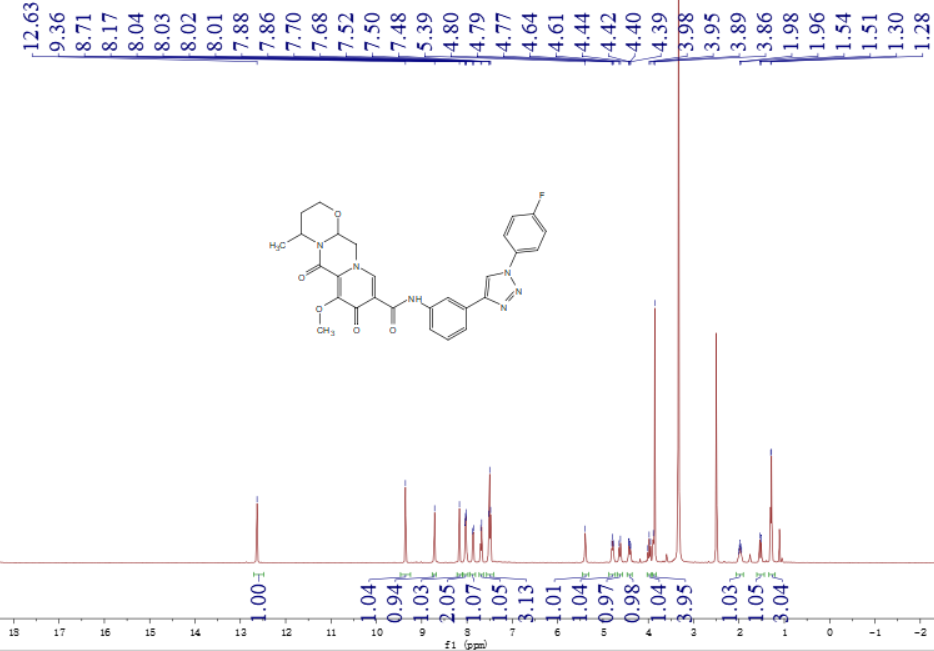


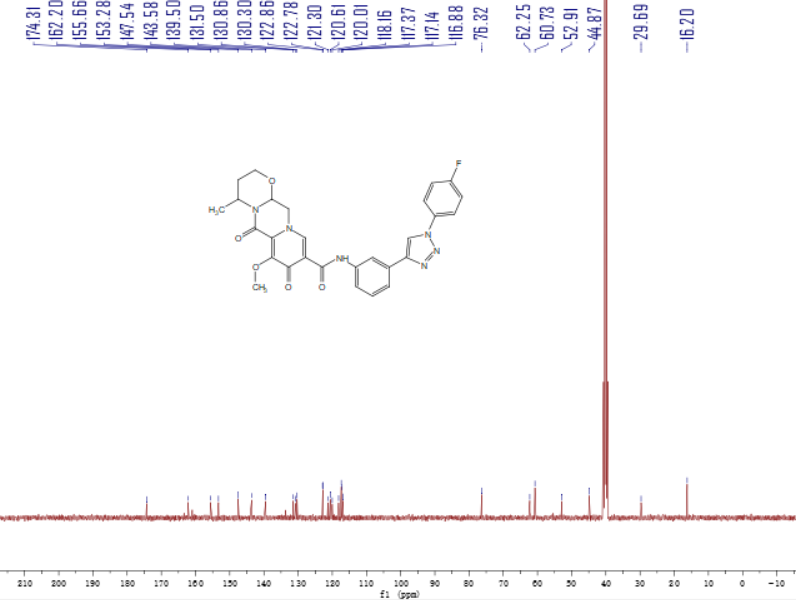


**Compound** 4e：Pure 98.7%. [white solid](javascript:;), HR-MS(ESI): Calcd. C30H30N6O5 [M+H]^+^ *m/z*: 555.2356, found: 555.2369. m.p. 176-179 ^o^C. ^1^H NMR (400MHz, DMSO-d_6_): 12.61 (s, 1H), 9.01 (s, 1H), 8.72 (s, 1H), 8.22 (s, 1H), 7.84 (d, *J*=8.0Hz, 1H), 7.69 (d, *J*=8.0Hz, 1H), 7.56 (dd, *J_1_*=8.0Hz, *J_2_*=8.0Hz, 1H), 7.51-7.44 (m, 3H), 5.39 (s, 1H), 4.79 (t, *J_1_*=8.0Hz, *J_2_*=4.0Hz, 1H), 4.63 (d, *J*=12.0Hz, 1H), 4.44-4.39 (m, 1H), 3.98 (t, *J_1_*=12.0Hz, *J_2_*=8.0Hz, 1H), 3.89 (s, 1H), 3.86 (s, 3H), 2.55 (d, *J*=8.0Hz, 2H), 2.01-1.95 (m, 1H), 1.54 (d, *J*=16.0Hz, 1H), 1.30 (d, *J*=4.0Hz, 3H), 1.06 (t, *J_1_*=8.0Hz, *J_2_*=8.0Hz, 3H)；^13^C NMR (100MHz, DMSO-d_6_): 173.77, 161.68, 155.15, 152.75, 146.17, 143.07, 139.25, 138.96, 135.71, 131.18, 130.33, 130.21, 129.86, 126.96, 126.37, 123.58, 120.88, 119.38, 61.73, 60.21, 52.36, 44.33, 29.16, 23.79, 15.67, 14.81


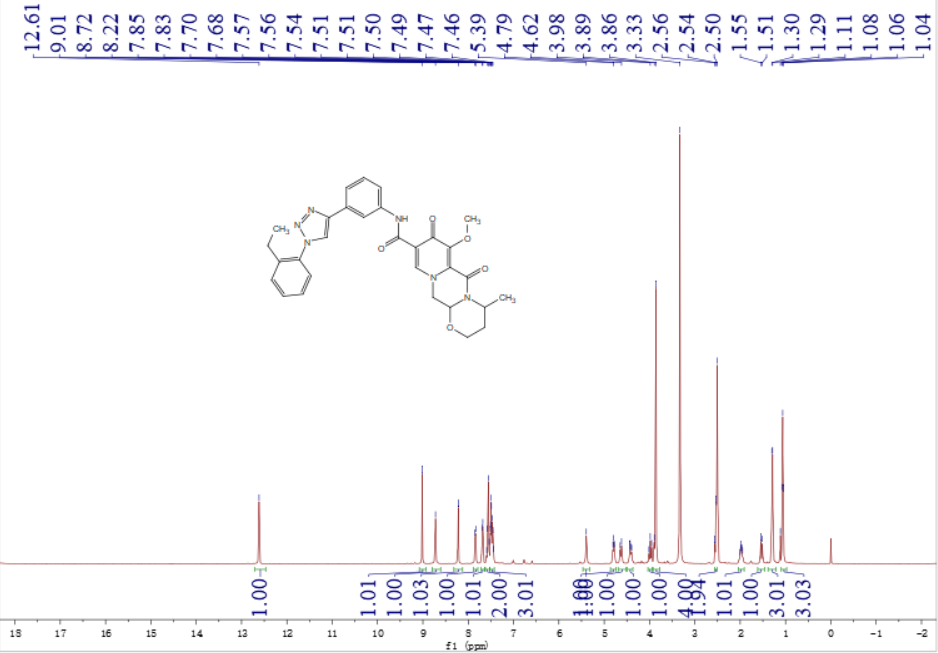


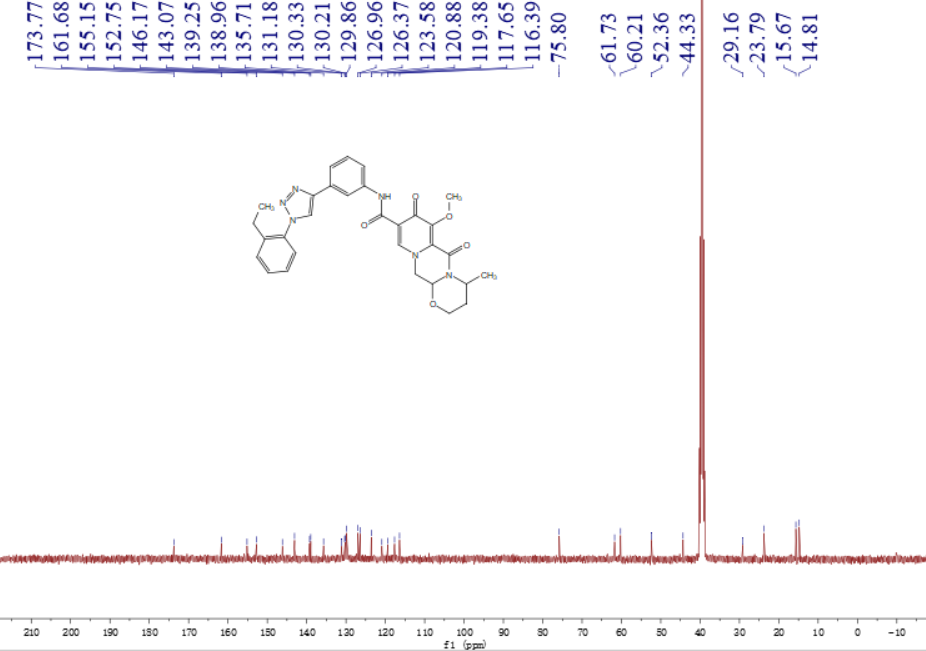


**Compound** 4f：Pure 96.4%. [white solid](javascript:;), HR-MS(ESI): Calcd. C29H28FN6O6 [M+H]^+^ *m/z*: 557.2149, found: 557.2148. m.p. 179-182 ^o^C. ^1^H NMR (400MHz, DMSO-d_6_): 12.64 (s, 1H), 9.40 (s, 1H), 8.72 (s, 1H), 8.17 (s, 1H), 7.88 (d, J=8.0Hz, 1H), 7.70 (d, J=8.0Hz, 1H), 7.59-7.48 (m, 4H), 7.09 (d, *J*=8.0Hz, 1H), 5.40 (s, 1H), 4.79 (t, *J_1_*=8.0Hz, *J_2_*=4.0Hz, 1H), 4.63 (d, *J*=12.0Hz, 1H), 4.42 (dd, *J_1_*=4.0Hz, *J_2_*=4.0Hz, 1H), 3.99 (t, *J_1_*=8.0Hz, *J_2_*=12.0Hz, 1H), 3.87 (d, *J*=12.0Hz, 7H), 2.03-1.94 (m, 1H), 1.53 (d, *J*=16.0Hz, 1H), 1.30 (d, *J*=4.0Hz, 3H); ^13^C NMR (100MHz, DMSO-d_6_): 173.77, 161.68, 160.20, 155.14, 152.74, 146.93, 143.06, 138.97, 137.63, 130.99, 130.83, 130.34, 129.76, 120.78, 119.87, 119.47, 117.63, 116.32, 114.40, 111.88, 105.50, 75.80, 61.72, 60.21, 55.61, 52.37, 44.33, 29.15, 15.66.


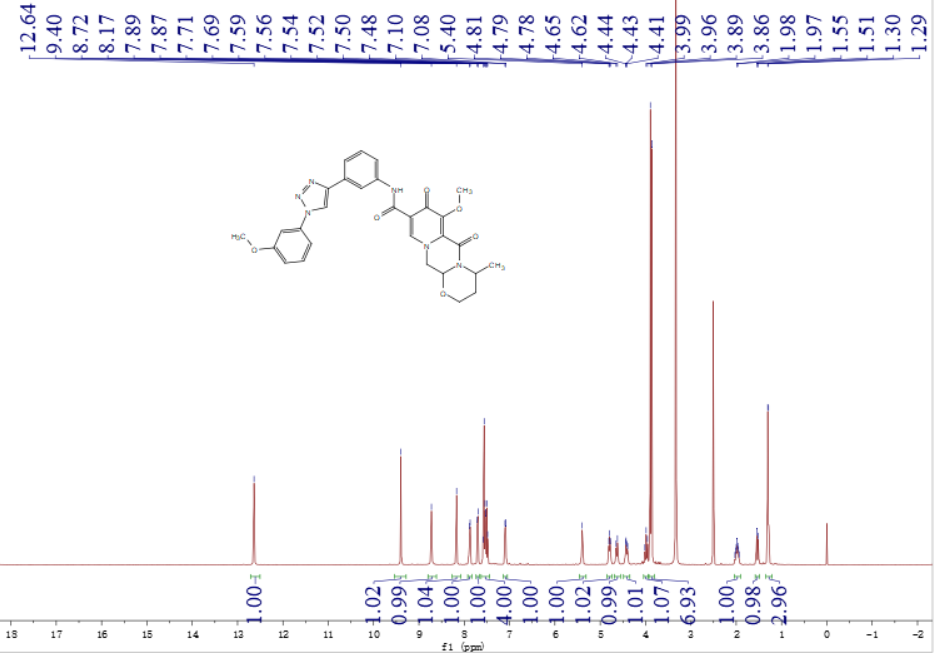


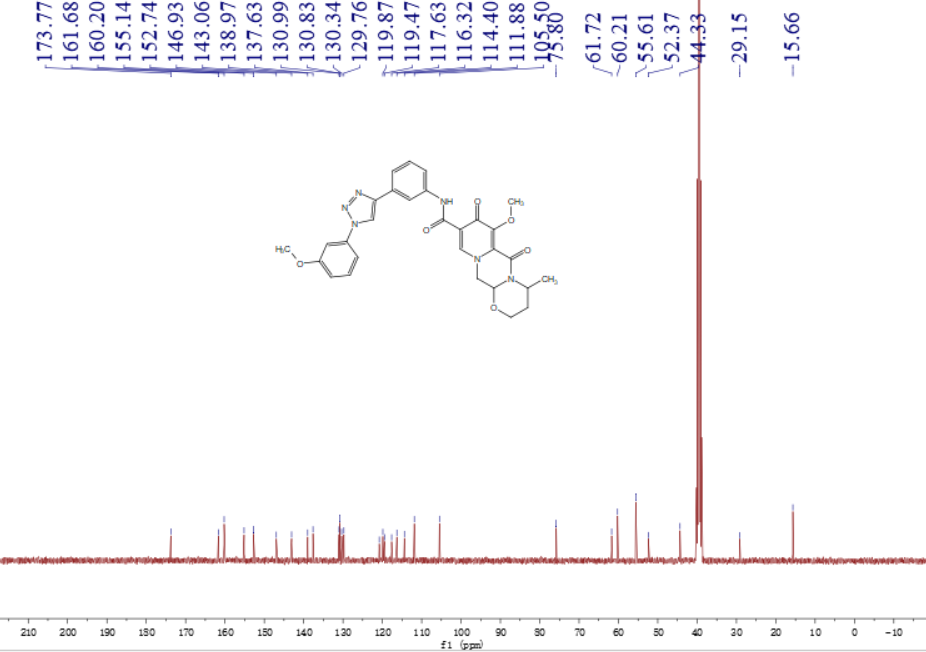


**Compound** 4g：Pure 98.9%. [yellow solid](javascript:;), HR-MS(ESI): Calcd. C29H25F3N6O5 [M+H]^+^ *m/z*: 595.1917, found: 595.1943. m.p. 203-207 ^o^C. ^1^H NMR (400MHz, DMSO-*d_6_*): 12.67 (s, 1H), 9.58 (s, 1H), 8.73 (s, 1H), 8.37-8.35 (m, 2H), 8.19 (s, 1H), 7.91-7.88 (m, 3H), 7.71 (d, *J*=8.0Hz, 1H), 7.52 (t, *J_1_*=8.0Hz, *J_2_*=4.0Hz, 1H), 5.40 (t, *J_1_*=4.0Hz, *J_2_*=4.0Hz, 1H), 4.81-4.78 (m, 1H), 4.66-4.62 (m, 1H), 4.45-4.40 (m, 1H), 3.99 (t, *J_1_*=12.0Hz, *J_2_*=12.0Hz, 1H), 3.90 (d, *J*=8.0Hz, 1H), 3.86 (s, 3H), 2.03-1.93 (m, 1H), 1.55-1.51 (m, 1H), 1.30 (d, *J*=4.0Hz, 3H); ^13^C NMR (100MHz, DMSO-d_6_): 174.29, 162.20, 155.65, 153.24, 147.74, 143.58, 139.53, 137.57, 131.86, 131.29, 130.88, 130.35, 125.71, 125.45, 124.31, 122.75, 11.26, 120.67, 120.09, 118.11, 117.05, 116.84, 76.31, 62.23, 60.71, 52.88, 44.84, 29.67, 16.18


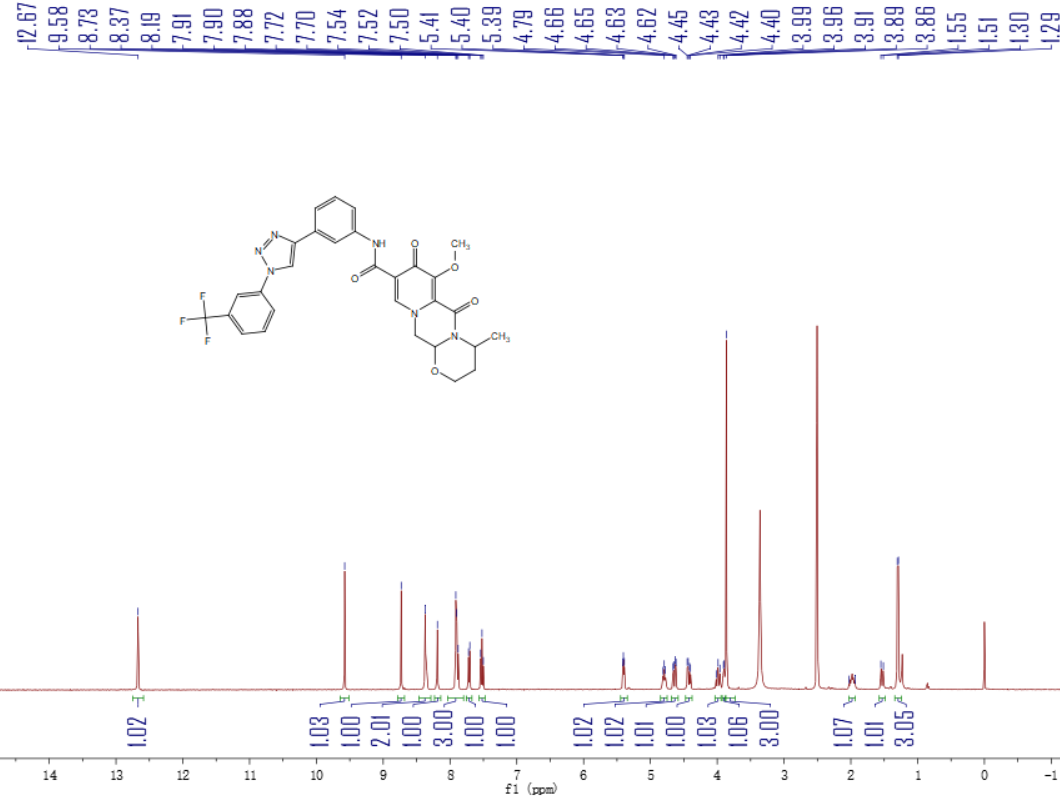


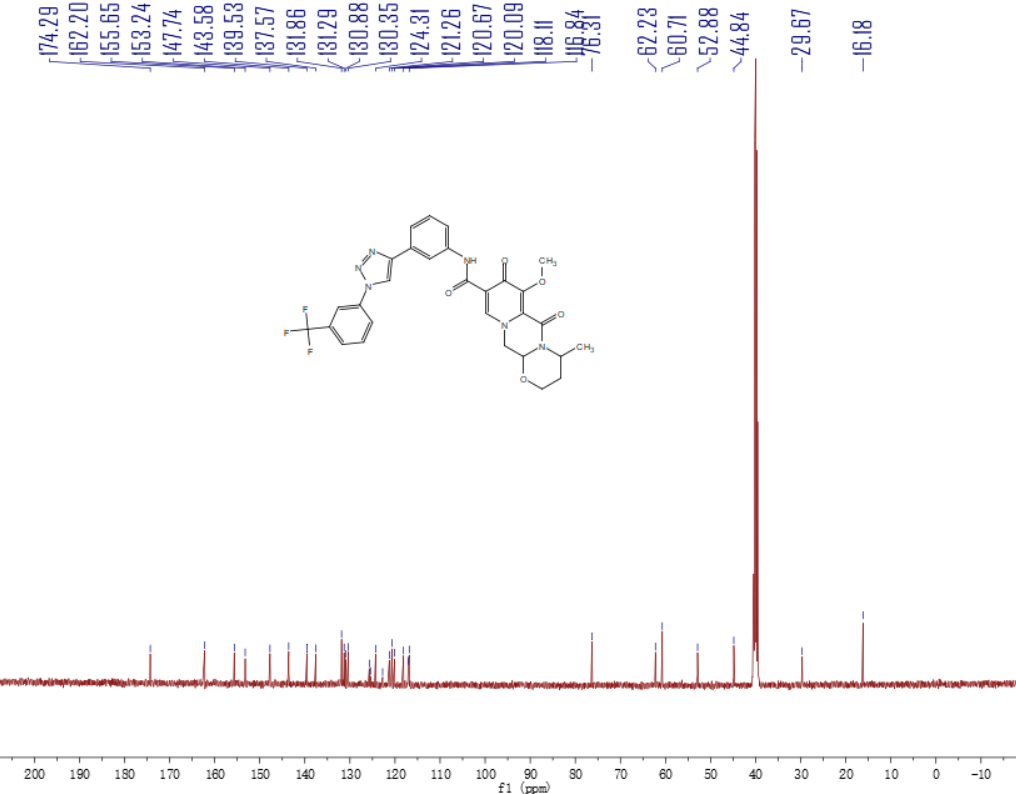


**Compound** 4h：Pure 98.3%. [yellow solid](javascript:;), HR-MS(ESI): Calcd. C30H24F6N6O5 [M+H]^+^ *m/z*: 663.1791, found: 663.1779. m.p. 206-209 ^o^C. ^1^H NMR (400MHz, DMSO-d_6_): 12.65 (s, 1H), 9.19 (s, 1H), 8.73 (s, 1H), 8.45 (s, 1H), 8.35-8.29 (m, 2H), 8.25 (s, 1H), 7.83 (d, *J*=8.0Hz, 1H), 7.69 (d, *J*=8.0Hz, 1H), 7.51 (t, *J_1_*=8.0Hz, *J_2_*=4.0Hz, 1H), 5.40 (t, *J_1_*=4.0Hz, *J_2_*=4.0Hz, 1H), 4.79 (t, *J_1_*=8.0Hz, *J_2_*=8.0Hz, 1H), 4.66-4.61 (m, 1H), 4.45-4.40 (m, 1H), 3.99 (t, *J_1_*=12.0Hz, *J_2_*=12.0Hz, 1H), 3.90 (s, 1H), 3.85 (s, 3H), 2.02-1.93 (m, 1H), 1.55-1.51 (m, 1H), 1.29 (d, *J*=4.0Hz, 3H); ^13^C NMR (100MHz, DMSO-d_6_): 174.28, 162.22, 155.65, 153.24, 146.87, 143.59, 139.55, 135.62, 134.56, 134.23, 131.14, 130.87, 130.38, 129.79, 128.67, 127.10, 125.16, 124.51, 123.96, 121.35, 120.12, 118.11, 116.93, 76.30, 62.22, 60.69, 52.86, 44.83, 29.67, 16.17.


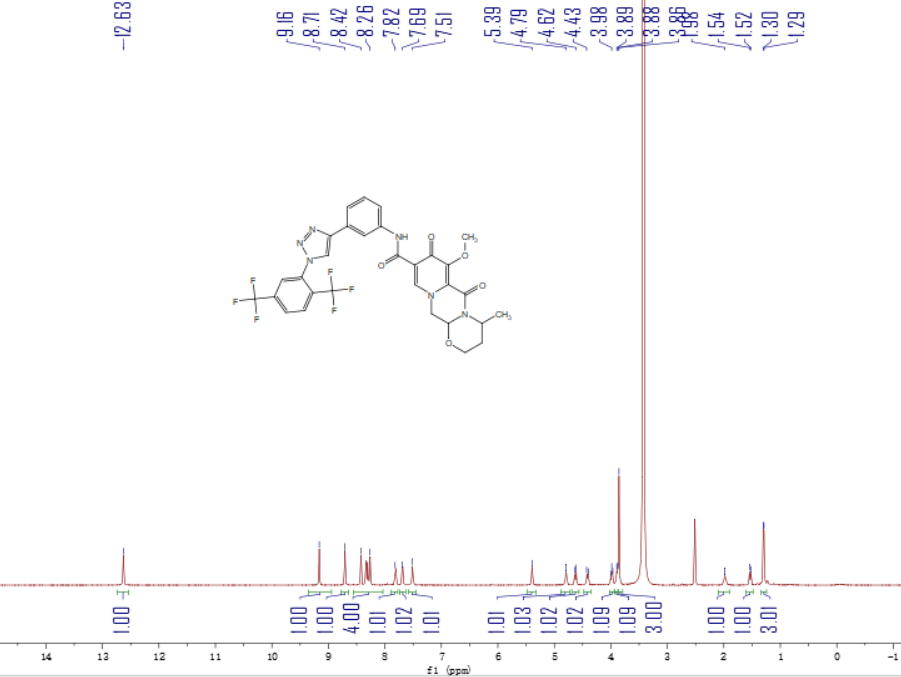


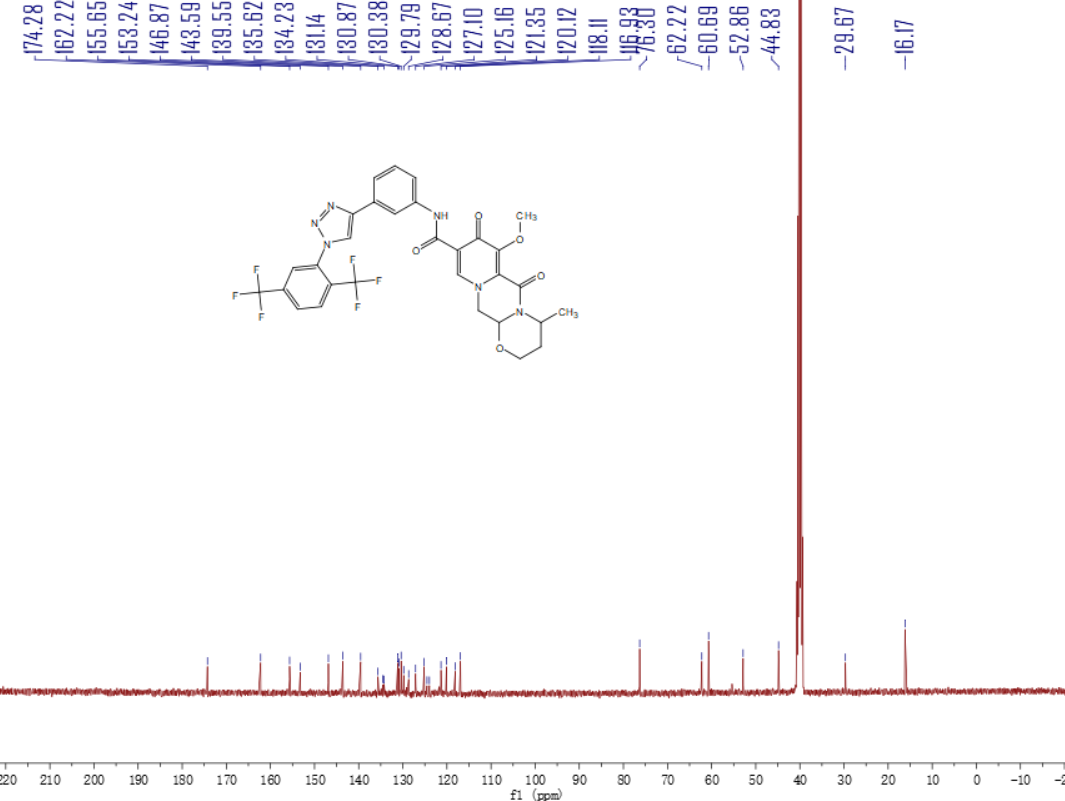


**Compound** 4i：Pure 98.1%. [white solid](javascript:;), HR-MS(ESI): Calcd. C29H28N6O6 [M+H]^+^ *m/z*: 557.2149, found: 557.2154. m.p. 177-180 ^o^C. ^1^H NMR (400MHz, DMSO-d_6_): 12.62 (s, 1H), 8.98 (s, 1H), 8.68 (s, 1H), 8.21 (s, 1H), 7.82-7.81 (m, 1H), 7.69 (d, *J*=8.0Hz, 2H), 7.59-7.55 (m, 1H), 7.48 (t, *J_1_*=8.0Hz, *J_2_*=8.0Hz, 1H), 7.39 (d, *J*=8.0Hz, 1H), 7.18 (t, *J_1_*=8.0Hz, *J_2_*=8.0Hz, 1H), 5.46-5.39 (m, 1H), 4.73-4.61 (m, 1H), 4.31-4.24 (m, 1H), 4.09-3.99 (m, 3H), 3.90 (s, 3H), 3.87 (d, *J*=8.0Hz, 3H), 2.29-2.18 (s, 1H), 1.99-1.88 (m, 1H), 1.41-1.28 (m, 3H); ^13^C NMR (100MHz, CDCl_3_): 161.73, 151.19, 146.98, 142.19, 141.99, 138.77, 131.48, 130.05, 129.57, 126.45, 125.53, 122.02, 121.77, 121.25, 120.20, 119.38, 117.68, 112.32, 99.99, 61.32, 61.20, 56.10, 55.27, 45.43, 26.68, 19.98.


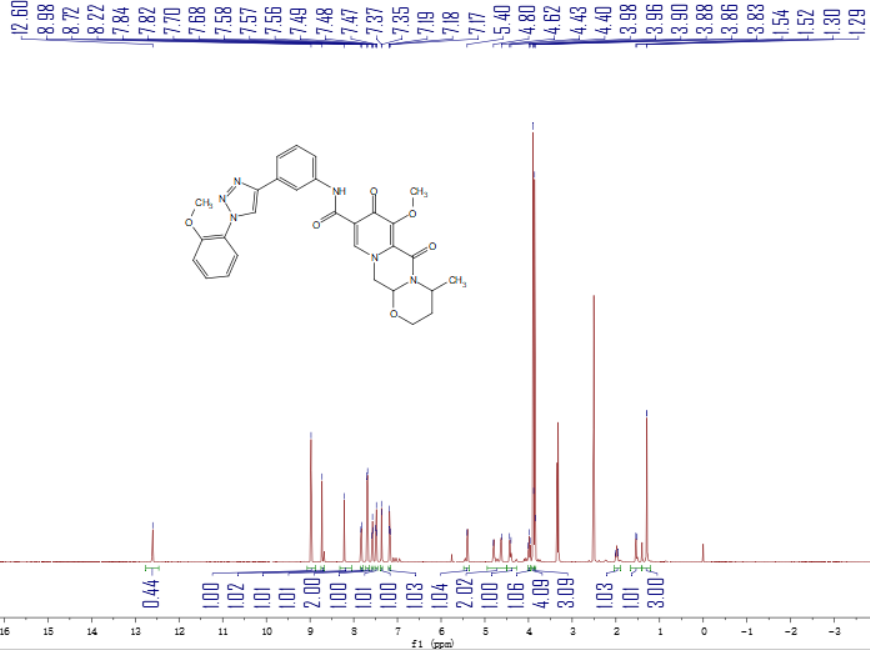


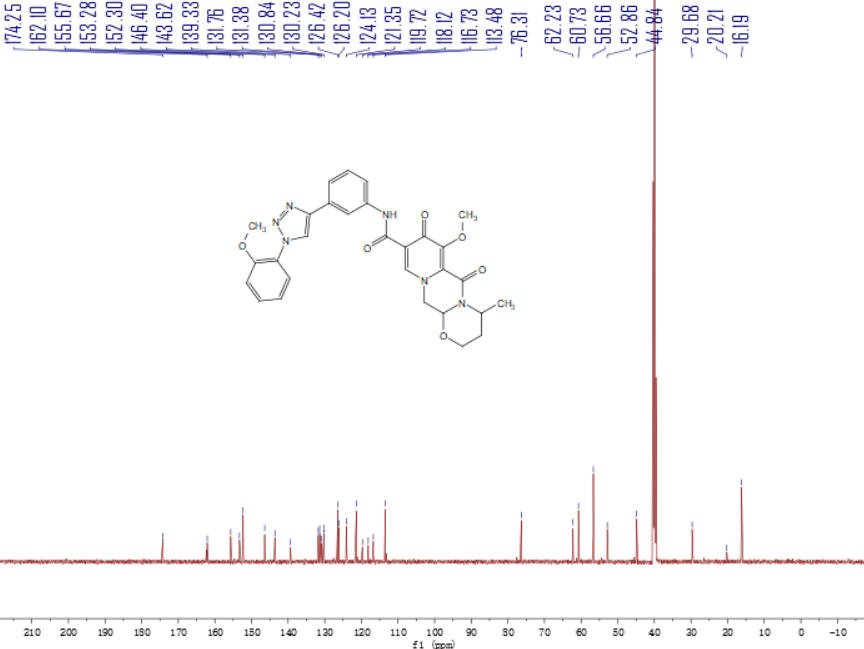


**Compound** 4j：Pure 97.7%. [white solid](javascript:;), HR-MS(ESI): Calcd. C28H25BrN6O5 [M+H]^+^ *m/z*: 605.1148, found: 605.1177. m.p. 175-178 ^o^C. ^1^H NMR (600MHz, DMSO-d_6_): 12.61 (s, 1H), 9.09 (s, 1H), 8.71 (s, 1H), 8.25-8.21 (m, 1H), 7.98 (dd, *J_1_*=6.0Hz, *J_2_*=12.0Hz, 1H), 7.87-7.48 (m, 6H), 5.40 (d, *J*=12.0Hz, 1H), 4.79 (t, *J_1_*=6.0Hz, *J_2_*=6.0Hz, 1H), 4.64-4.61 (m, 1H), 4.42 (dd, *J_1_*=6.0Hz, *J_2_*=6.0Hz, 1H), 3.98 (t, *J_1_*=12.0Hz, *J_2_*=12.0Hz, 1H), 3.90-3.88 (m, 1H), 3.86 (ds, 3H), 2.01-1.95 (m, 1H), 1.54-1.52 (m, 1H), 1.29 (d, J=6.0Hz, 3H).^13^C NMR (150MHz, DMSO-d_6_): 174.28, 162.21, 155.67, 153.26, 146.64, 143.59, 139.47, 136.65, 134.15, 132.58, 131.46, 130.86, 130.43, 129.50, 129.17, 124.38, 120.46, 120.00, 119.34, 118.13, 116.88, 76.30, 62.25, 60.75, 52.89, 44.87, 29.67, 16.18


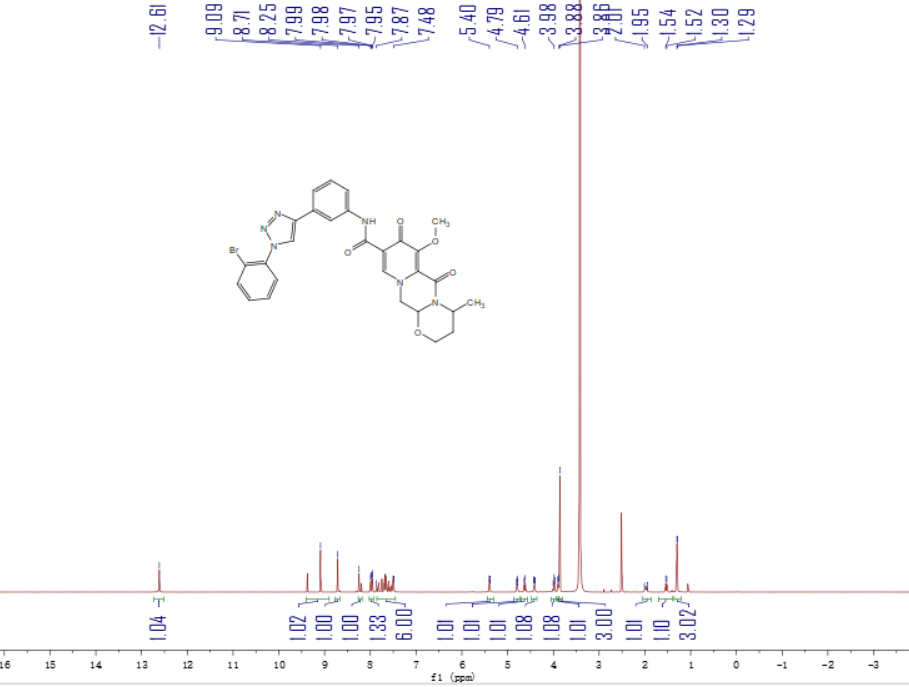


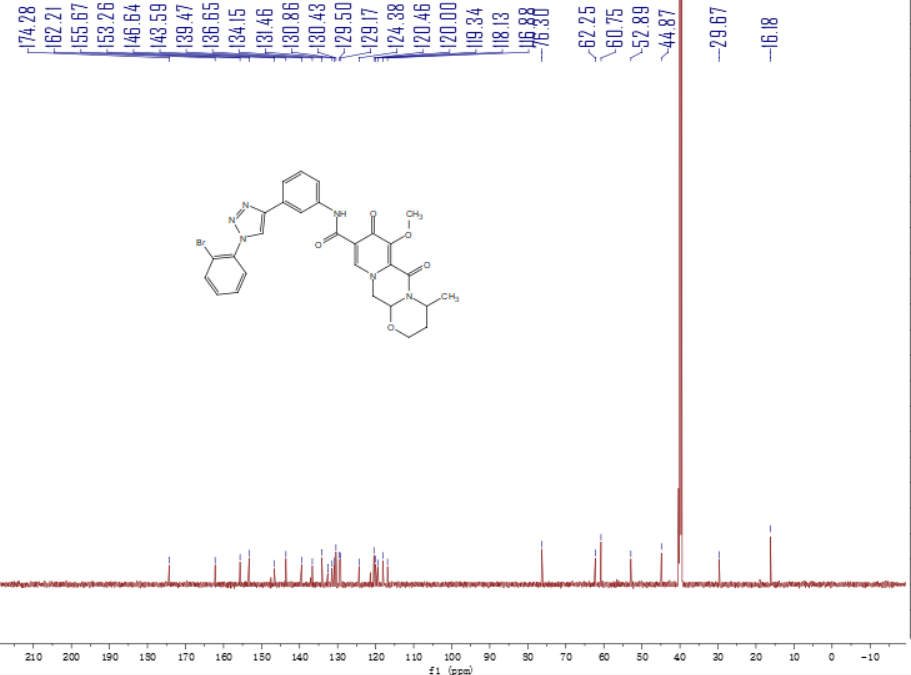


**Compound** 4k：Pure 96.4%. [white solid](javascript:;), HR-MS(ESI): Calcd. C28H25ClN6O5 [M+H]^+^ *m/z*: 561.1653, found: 561.1699. m.p. 150-153 ^o^C. ^1^H NMR (400MHz, DMSO-d_6_): 12.65-12.63 (m, 1H), 9.13 (s, 1H), 8.73-8.68 (m, 1H), 8.23 (s, 1H), 7.84-7.79 (m, 3H), 7.70-7.61 (m, 3H), 7.52-7.42 (m, 2H), 5.46-5.39 (m, 1H), 4.74-4.61 (m, 1H), 4.30-4.25 (m, 1H), 4.09-3.99 (m, 2H), 3.88-3.85 (m, 3H), 2.29-2.16 (m, 1H), 1.98-1.88 (m, 1H), 1.41-1.28 (m, 3H); ^13^C NMR (100MHz, DMSO-d_6_): 174.22, 162.28, 155.66, 154.62, 153.24, 152.23, 146.70, 143.63, 139.49, 134.99, 132.26, 131.09, 130.31, 129.01, 126.69, 124.38, 121.38, 120.01, 117.94, 116.90, 77.53, 60.73, 52.85, 45.42, 29.68, 26.41, 20.21, 16.19.


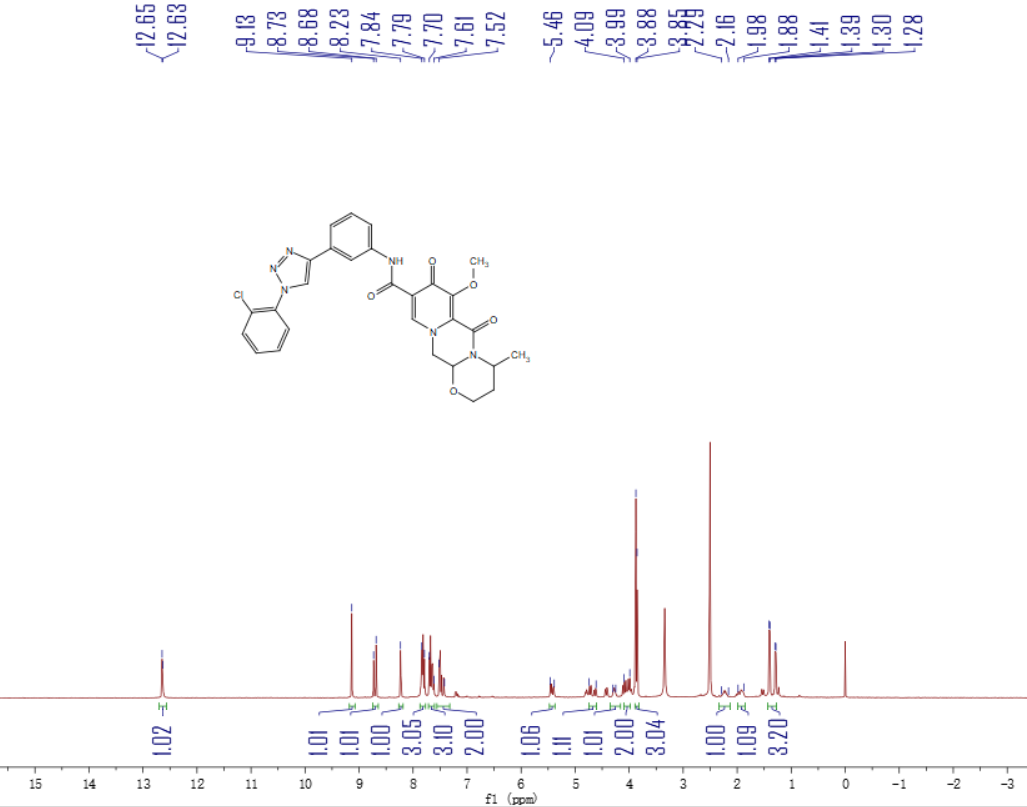


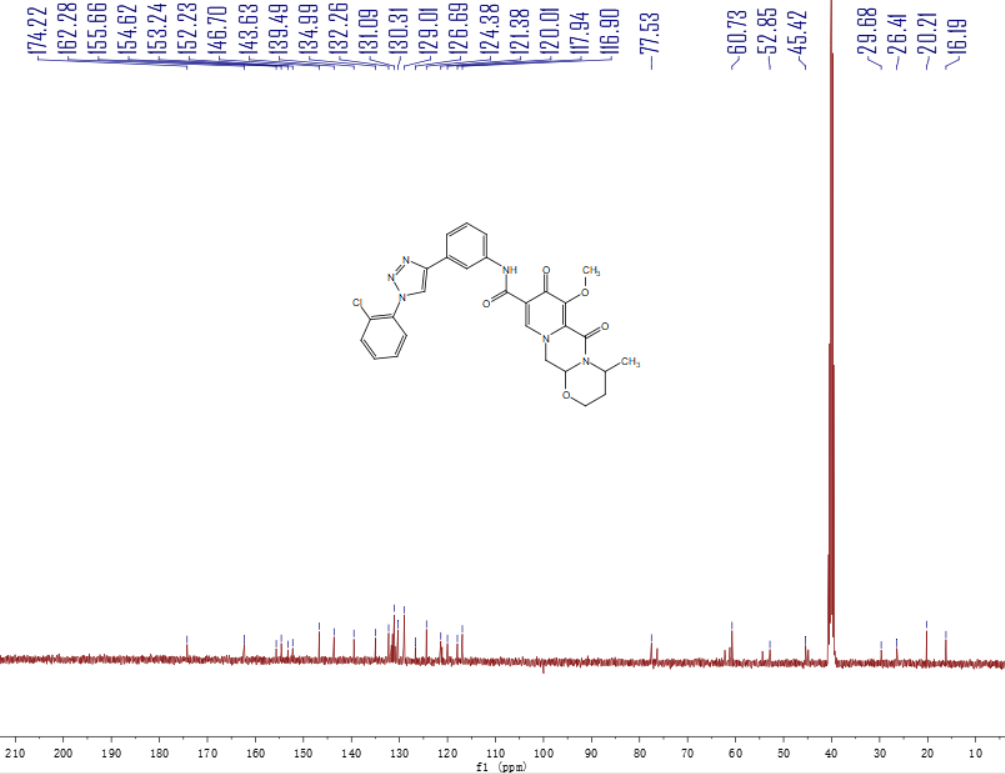


**Compound** 4l：Pure 98.2%. [white solid](javascript:;), HR-MS(ESI): Calcd. C28H25IN6O5 [M+H]^+^ *m/z*: 653.1009, found: 653.1086. m.p. 248-251 ^o^C. ^1^H NMR (400MHz, DMSO-d_6_): 12.65-12.63 (m, 1H), 9.41 (s, 1H), 8.73 (s, 1H), 8.18 (s, 1H), 7.99 (d, *J*=8.0Hz, 1H), 7.90-7.83 (m, 1H), 7.72-7.63 (m, 3H), 7.55-7.47 (m, 2H), 5.40 (t, *J_1_*=4.0Hz, *J_2_*=4.0Hz, 1H), 4.82-4.76 (m, 1H), 4.66-4.62 (m, 1H), 4.45-4.40 (m, 1H), 4.01-3.96 (m, 1H), 3.89 (d, *J*=8.0Hz, 1H), 3.86-3.85 (m, 3H), 2.01-1.93 (m, 1H), 1.54-1.51 (m,1H), 1.30 (d, *J*=4.0Hz, 3H). ^13^C NMR (100MHz, DMSO-d_6_): 174.29, 162.20, 155.66, 153.24, 147.50, 143.59, 140.26, 139.50, 137.12, 131.54, 130.88, 130.42, 130.30, 129.20, 121.29, 120.44, 120.35, 119.96, 118.13, 116.84, 76.31, 62.23, 60.72, 55.38, 52.86, 44.84, 29.68, 16.19


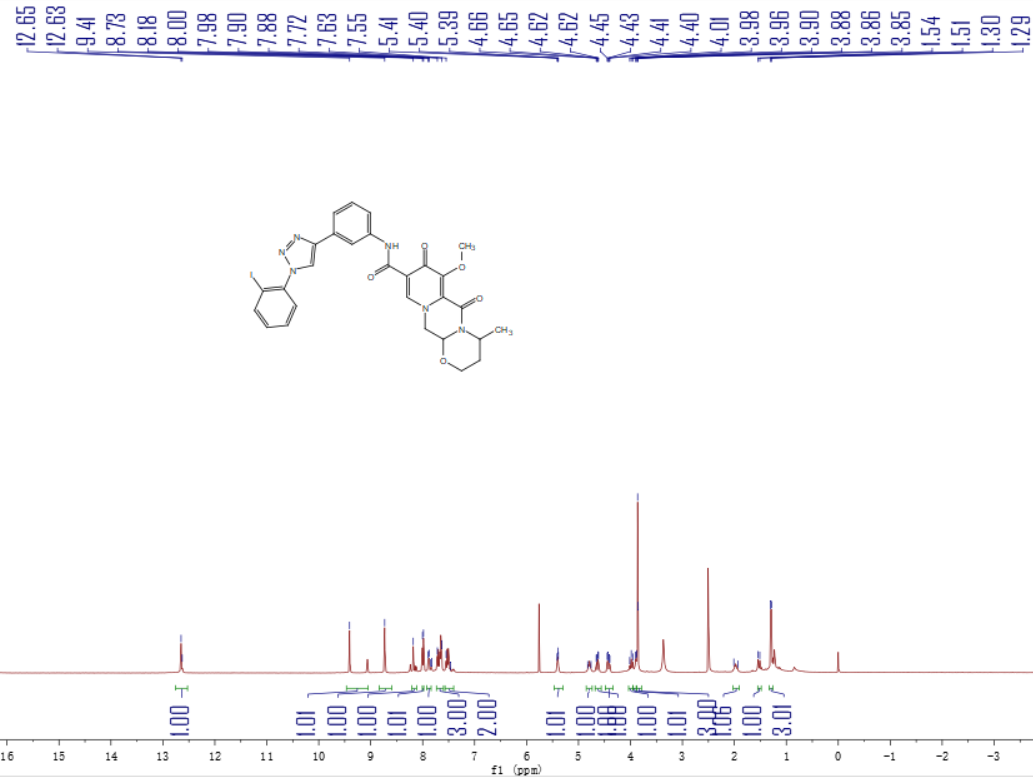


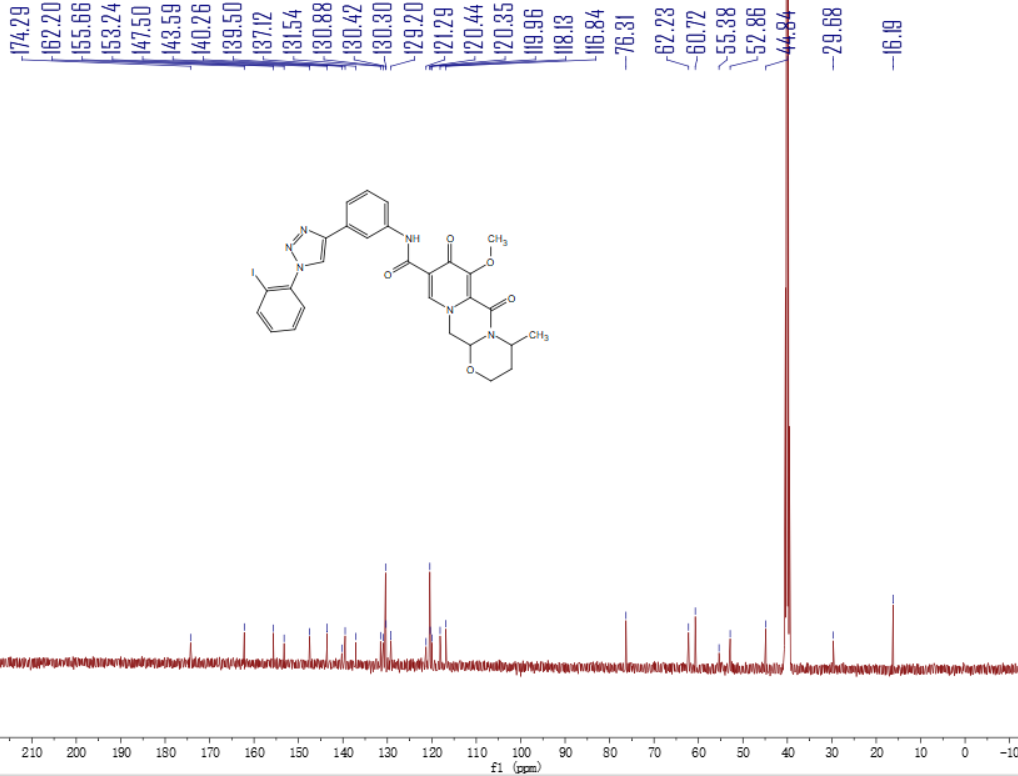


**Compound** 4m：Pure 97.8%. [white solid](javascript:;), HR-MS(ESI): Calcd. C29H27BrN6O5 [M+H]^+^ *m/z*: 619.1305, found: 619.1357. m.p. 200-203 ^o^C. ^1^H NMR (400MHz, DMSO-d_6_): 12.58 (s, 1H), 8.71 (s, 1H), 8.68 (s, 1H), 8.15 (s, 1H), 7.77-7.71 (m, 2H), 7.61 (d, J=4.0Hz, 1H), 7.46-7.42 (m, 2H), 7.36-7.32 (m, 1H), 7.27 (d, *J*=4.0Hz, 1H), 5.75 (s, 2H), 5.40-5.38 (m, 1H), 4.82-4.77 (m, 1H), 4.65-4.60 (m, 1H), 4.41 (d, *J_1_*=4.0Hz, *J_2_*=8.0Hz, 1H), 4.01-3.95 (m, 1H), 3.90-3.89 (m, 1H), 3.85 (s, 3H), 2.01-1.93 (m, 1H), 1.54-1.50 (m, 1H), 1.29 (d, *J*=8.0Hz, 3H). ^13^C NMR (100MHz, DMSO-d_6_): 174.25, 162.16, 155.65, 153.65, 153.24, 146.66, 143.57, 139.39, 135.23, 133.40, 131.85, 131.06, 130.93, 130.83, 130.16, 128.82, 123.39, 122.70, 121.20, 119.67, 118.14, 116.74, 76.30, 62.22, 60.71, 60.59, 53.63, 52.86, 44.83, 29.67, 16.18


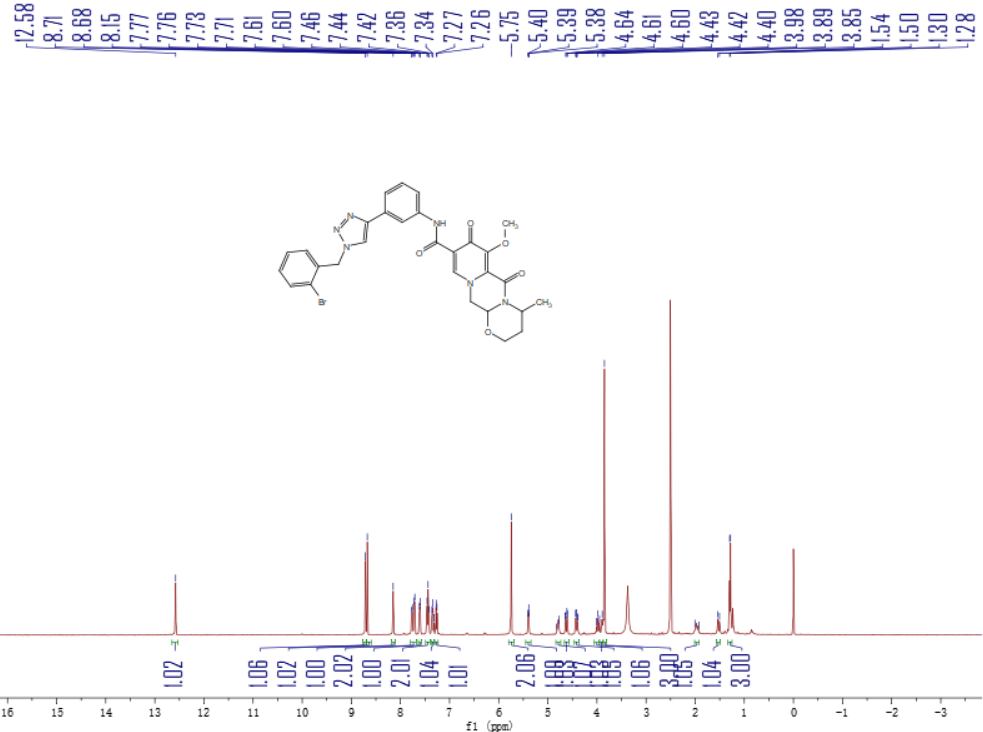


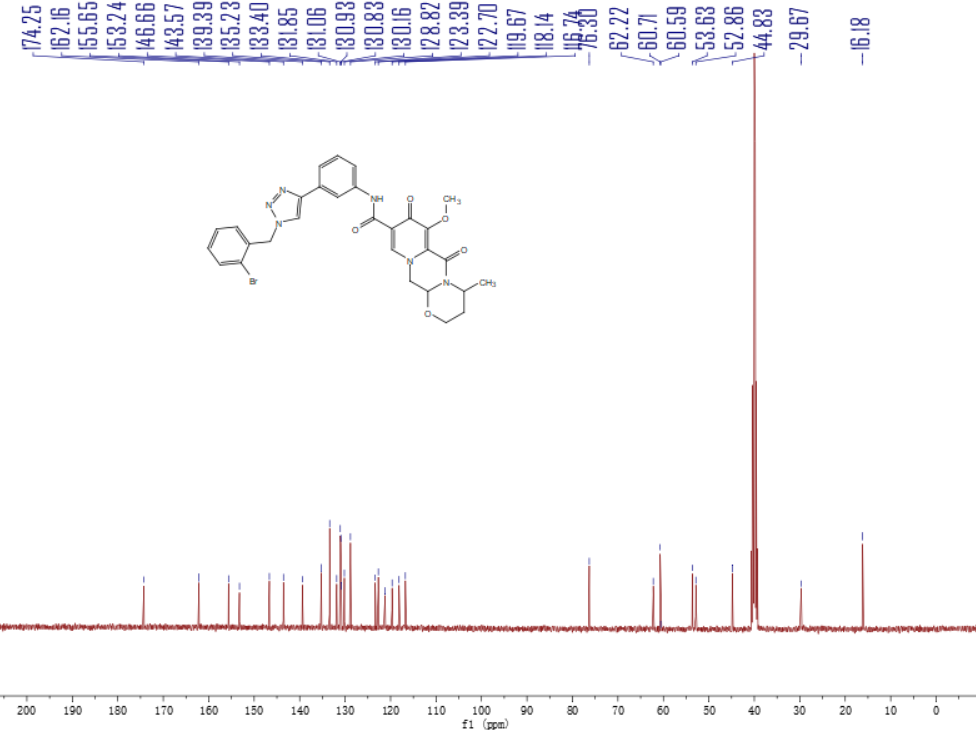


**Compound** 4n：Pure 98.9%. [white solid](javascript:;), HR-MS(ESI): Calcd. C29H27BrN6O5 [M+H]^+^ *m/z*: 619.1305, found: 619.1340. m.p. 127-130 ^o^C. ^1^H NMR (400MHz, DMSO-d_6_): 12.59 (s, 1H), 8.75 (s, 1H), 8.71 (s, 1H), 8.14 (s, 1H), 7.76 (d, *J*=8.0Hz,1H), 7.63-7.55 (m, 3H), 7.44 (t, *J_1_*=8.0 Hz, *J_2_*=8.0 Hz, 1H), 7.38 (d, *J*=4.0 Hz, 2H), 5.68 (s, 2H), 5.41-5.38 (m, 1H), 4.80-4.77 (m, 1H), 4.65-4.60 (m, 1H), 4.41 (d, *J_1_*=4.0 Hz, *J_2_*=4.0 Hz, 1H), 4.01-3.95 (m, 1H), 3.90-3.89 (m, 1H), 3.85 (s, 3H), 2.02-1.93 (m, 1H), 1.54-1.51 (m, 1H), 1.29 (d, *J*=8.0 Hz, 3H). ^13^C NMR (100MHz, DMSO-d_6_): 174.26, 162.16, 155.66, 153.24, 146.90, 143.57, 139.40, 139.03, 131.85, 131.58, 131.52, 131.28, 130.86, 130.20, 127.61, 122.48, 122.36, 121.16, 119.66, 118.13, 116.71, 76.30, 62.23, 60.70, 52.86, 52.71, 44.84, 29.68, 16.19


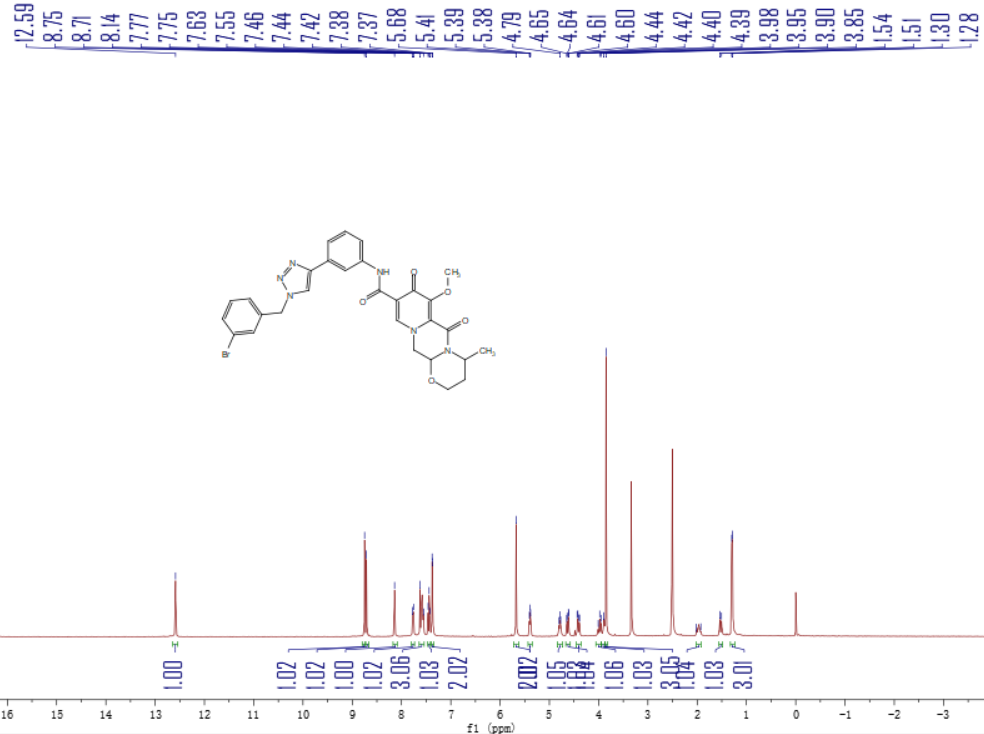


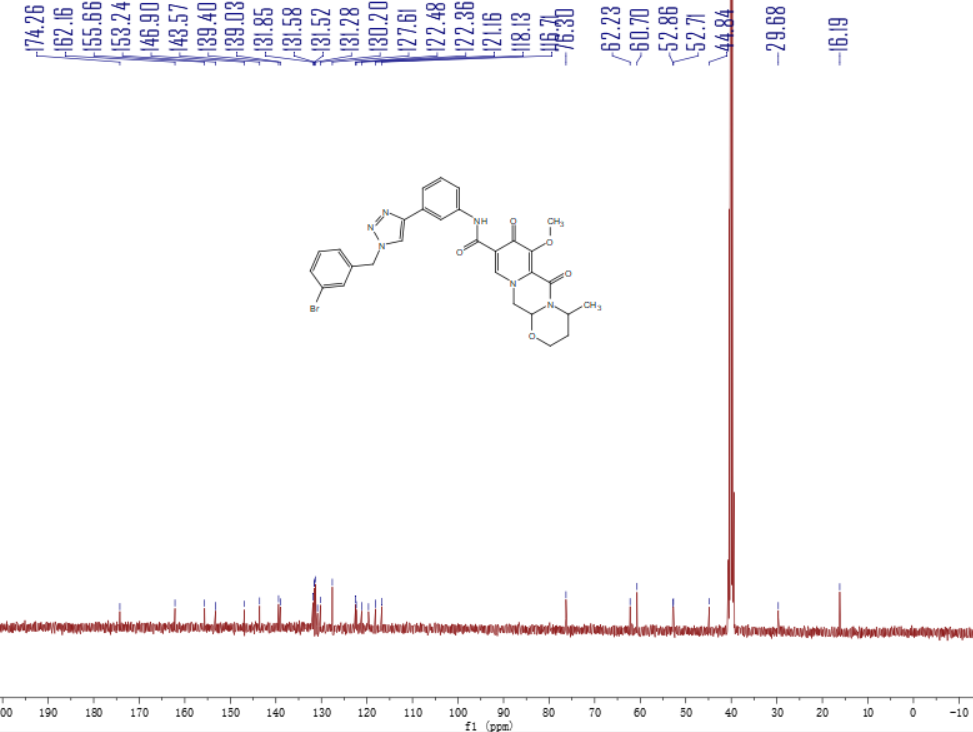

Supplement: Supplementary file 1 [file DataSheet2.docx]
