# Supplementary material for: Design, synthesis and antitumour activity evaluation of novel dolutegravir derivatives
Source: Front Pharmacol. 2023 Aug 7;14:1238587. doi: 10.3389/fphar.2023.1238587 (PMC10440426; doi:10.3389/fphar.2023.1238587)

**LC3 western blot analysis of 4b.**


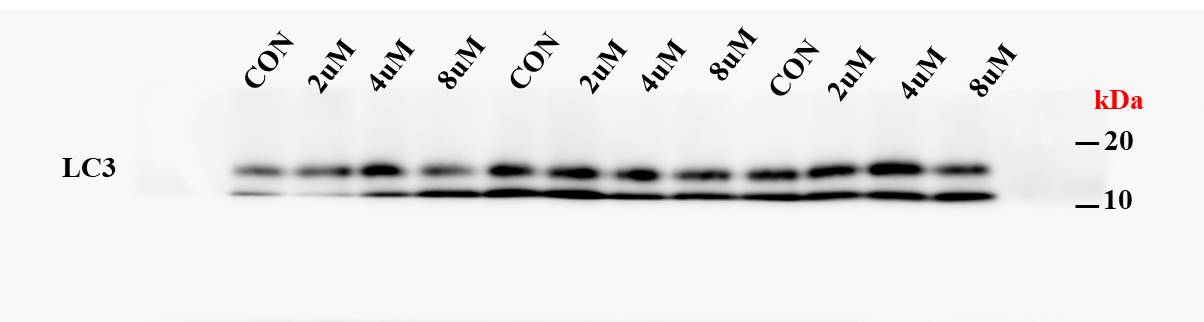


**Caspase3 western blot analysis of 4b.**


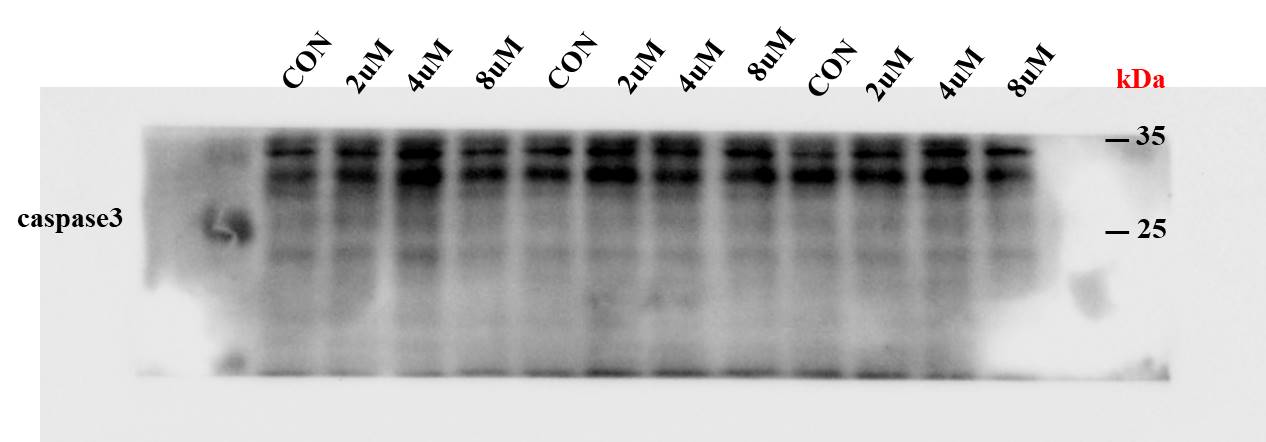


**cyclinD western blot analysis of 4b.**


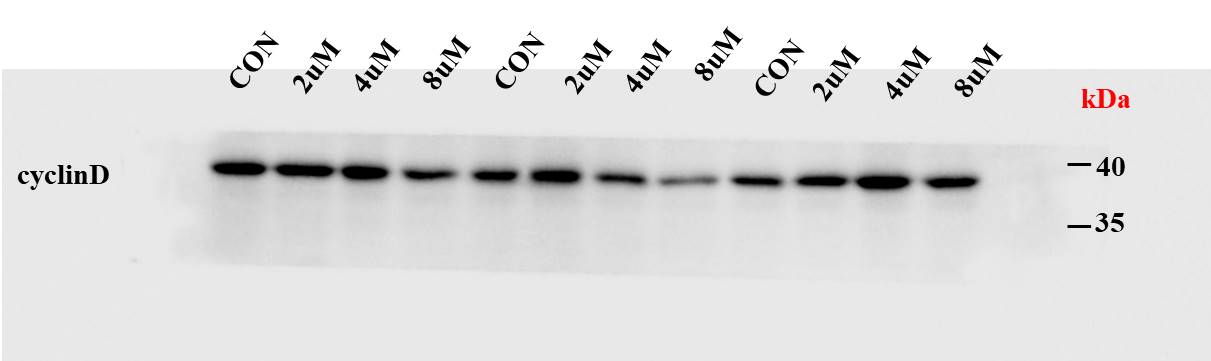


**cyclinE western blot analysis of 4b.**


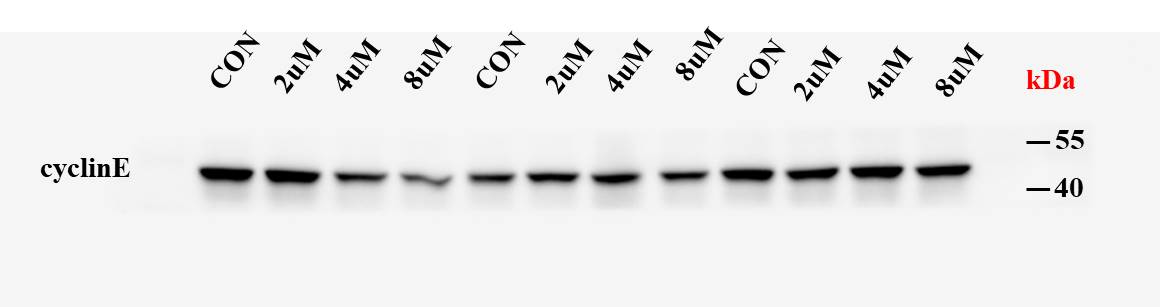


**catenin western blot analysis of 4b.**


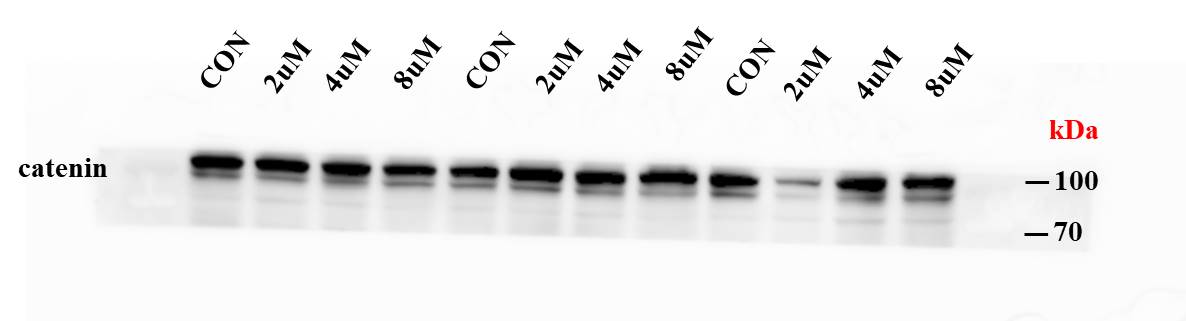


**γ-H2AX western blot analysis of 4b.**


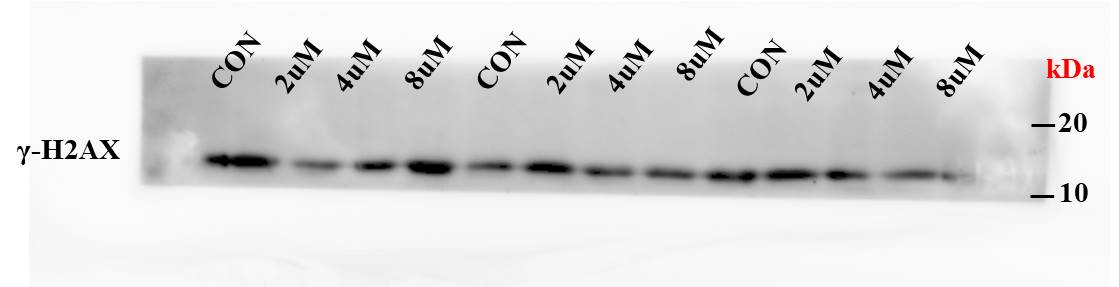


**PARP western blot analysis of 4b.**


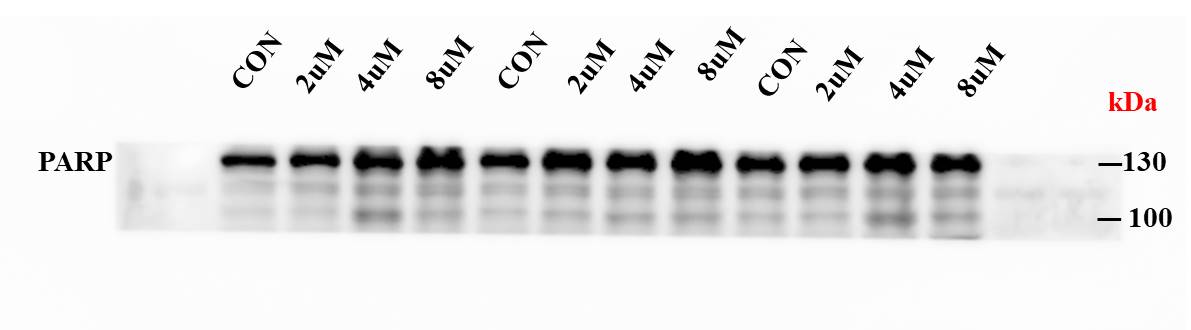


**ACTIN western blot analysis of 4b.**


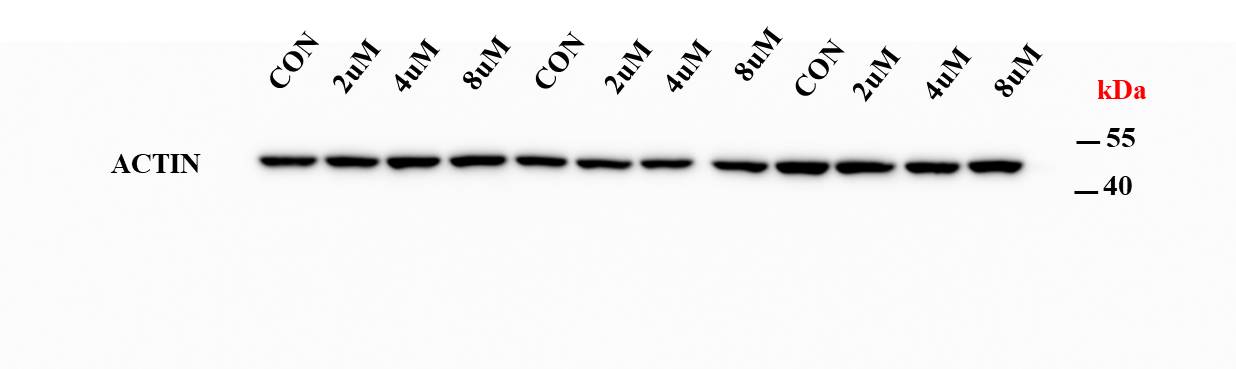


**LC3 western blot analysis of 4g.**


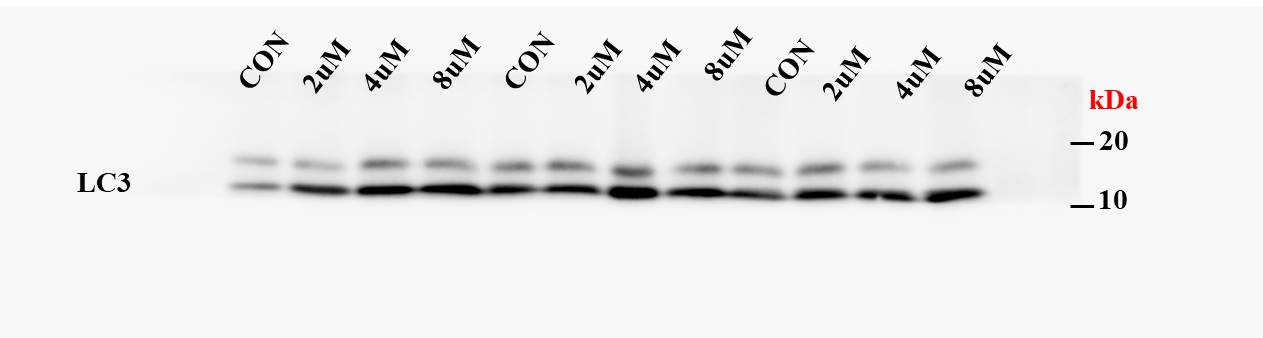


**Caspase3 western blot analysis of 4g.**


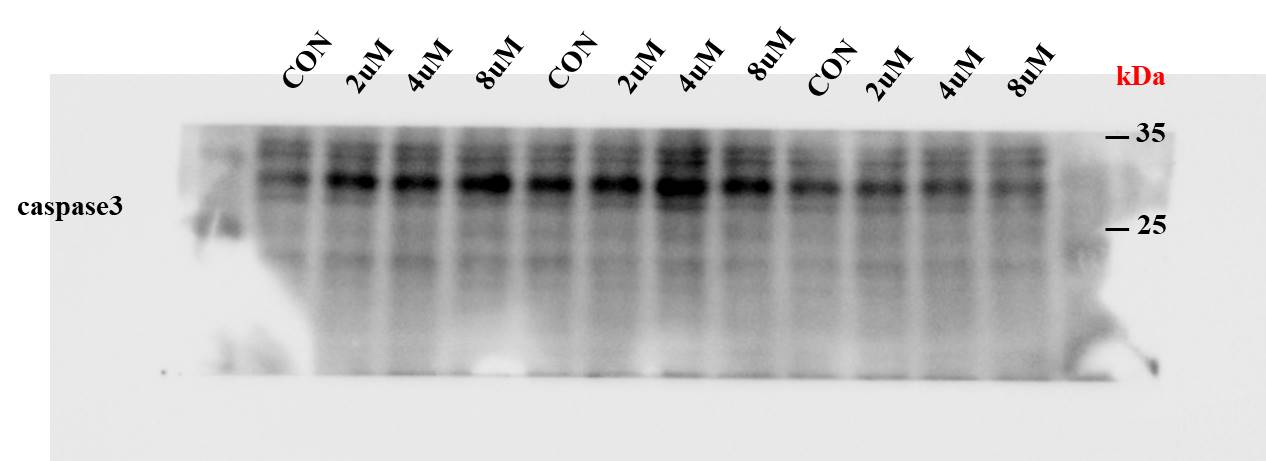


**cyclinD western blot analysis of 4g.**


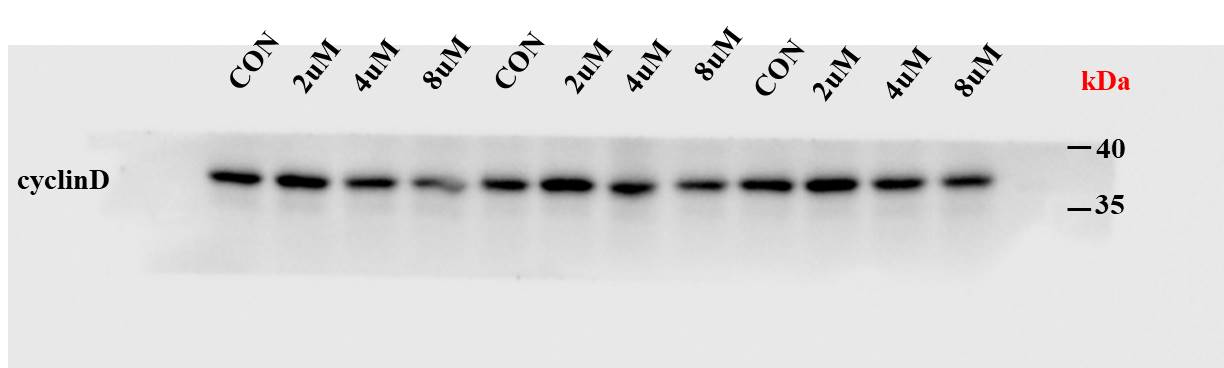


**cyclinE western blot analysis of 4g.**


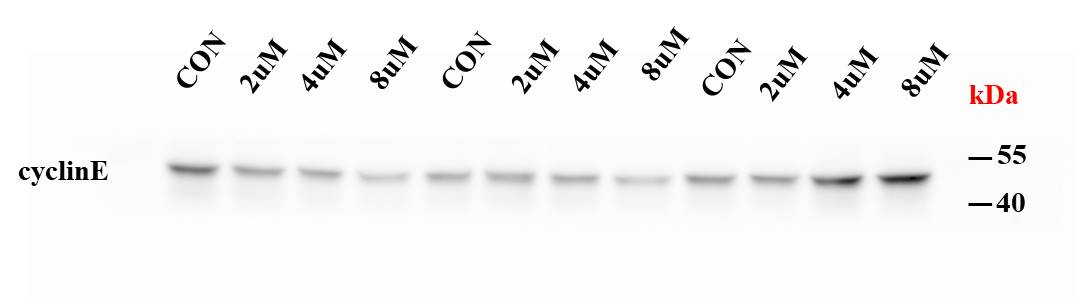


**catenin western blot analysis of 4g.**


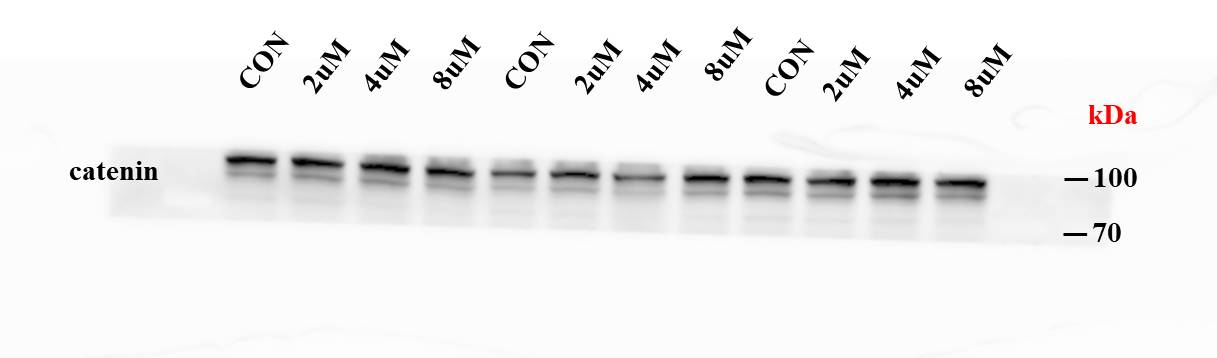


**γ-H2AX western blot analysis of 4g.**


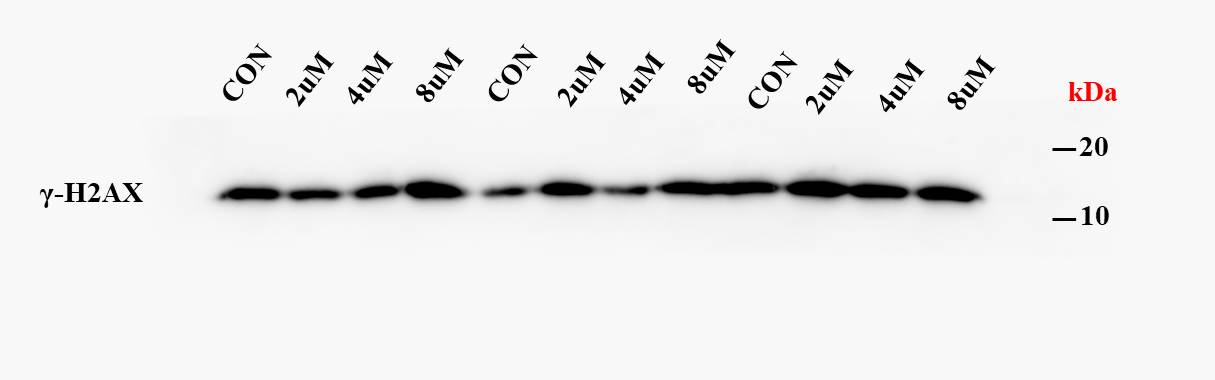


**PARP western blot analysis of 4g.**


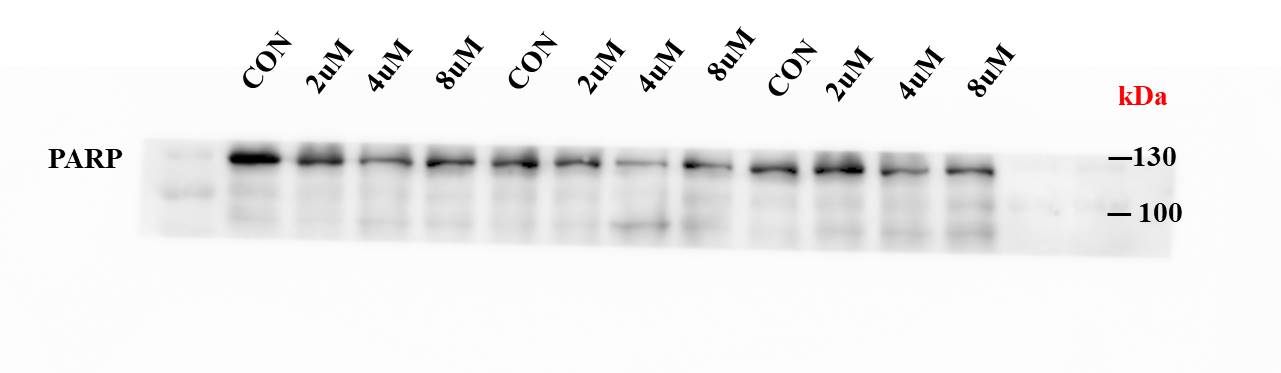


**ACTIN western blot analysis of 4g.**


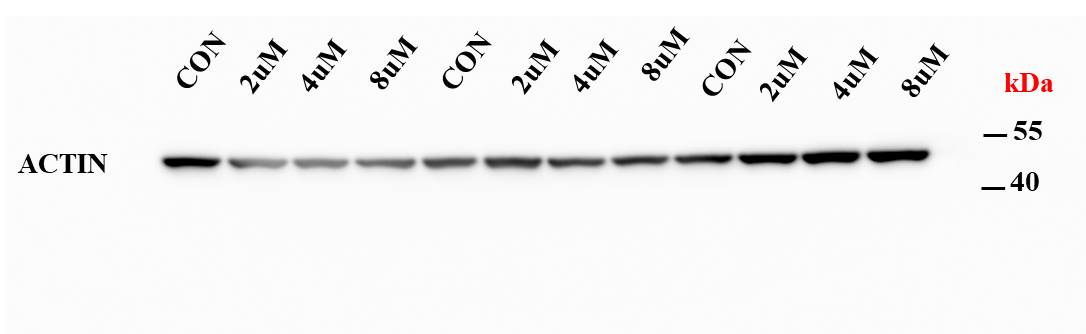

Supplement: Supplementary file 2 [file DataSheet1.docx]
